# Synchronized Expansion and Contraction of Olfactory, Vomeronasal, and Taste Receptor Gene Families in Hystricomorph Rodents

**DOI:** 10.1093/molbev/msae071

**Published:** 2024-04-23

**Authors:** Yoshihito Niimura, Bhim B Biswa, Takushi Kishida, Atsushi Toyoda, Kazumichi Fujiwara, Masato Ito, Kazushige Touhara, Miho Inoue-Murayama, Scott H Jenkins, Christopher Adenyo, Boniface B Kayang, Tsuyoshi Koide

**Affiliations:** Department of Veterinary Sciences, Faculty of Agriculture, University of Miyazaki, Miyazaki, Japan; Mouse Genomics Resource Laboratory, National Institute of Genetics, Mishima, Japan; Department of Genetics, SOKENDAI (The Graduate University for Advanced Studies), Shizuoka, Japan; Curatorial Division, Museum of Natural and Environmental History, Shizuoka, Japan; Present address: College of Bioresource Sciences, Nihon University, Fujisawa, Japan; Comparative Genomics Laboratory, National Institute of Genetics, Shizuoka, Japan; Mouse Genomics Resource Laboratory, National Institute of Genetics, Mishima, Japan; Department of Applied Biological Chemistry, Graduate School of Agricultural and Life Sciences, The University of Tokyo, Tokyo, Japan; Department of Applied Biological Chemistry, Graduate School of Agricultural and Life Sciences, The University of Tokyo, Tokyo, Japan; Wildlife Research Center, Kyoto University, Kyoto, Japan; Wildlife Research Center, Kyoto University, Kyoto, Japan; Present address: Biosphere Informatics Laboratory, Department of Social Informatics, Graduate School of Informatics, Kyoto, Japan; Livestock and Poultry Research Centre, College of Basic and Applied Sciences, University of Ghana, Accra, Ghana; Department of Animal Science, College of Basic and Applied Sciences, University of Ghana, Accra, Ghana; Mouse Genomics Resource Laboratory, National Institute of Genetics, Mishima, Japan; Department of Genetics, SOKENDAI (The Graduate University for Advanced Studies), Shizuoka, Japan

**Keywords:** olfactory receptor, vomeronasal receptor, taste receptor, multigene family, pheromone, hystricomorph rodents

## Abstract

Chemical senses, including olfaction, pheromones, and taste, are crucial for the survival of most animals. There has long been a debate about whether different types of senses might influence each other. For instance, primates with a strong sense of vision are thought to have weakened olfactory abilities, although the oversimplified trade-off theory is now being questioned. It is uncertain whether such interactions between different chemical senses occur during evolution. To address this question, we examined four receptor gene families related to olfaction, pheromones, and taste: olfactory receptor (OR), vomeronasal receptor type 1 and type 2 (V1R and V2R), and bitter taste receptor (T2R) genes in Hystricomorpha, which is morphologically and ecologically the most diverse group of rodents. We also sequenced and assembled the genome of the grasscutter, *Thryonomys swinderianus*. By examining 16 available genome assemblies alongside the grasscutter genome, we identified orthologous gene groups among hystricomorph rodents for these gene families to separate the gene gain and loss events in each phylogenetic branch of the Hystricomorpha evolutionary tree. Our analysis revealed that the expansion or contraction of the four gene families occurred synchronously, indicating that when one chemical sense develops or deteriorates, the others follow suit. The results also showed that V1R/V2R genes underwent the fastest evolution, followed by OR genes, and T2R genes were the most evolutionarily stable. This variation likely reflects the difference in ligands of V1R/V2Rs, ORs, and T2Rs: species-specific pheromones, environment-based scents, and toxic substances common to many animals, respectively.

## Introduction

The chemical senses play a critical role in the survival of most mammals, enabling them to locate food, find mates and offspring, identify territories, and avoid potential dangers. Mammalian species rely on at least five different multigene families of G protein–coupled receptors (GPCRs) for chemosensation, including olfactory receptors (ORs), vomeronasal receptors, and taste receptors (Table 1; Nei et al. 2008; Niimura et al. 2020). Among these families, the OR gene family is notably the largest, reflecting the vast diversity of odor molecules present in the environment. The number of OR genes encoded in the genome varies significantly across species, with ∼400 in humans, ∼1,100 in mice, and ∼2,000 in African elephants (Niimura and Nei 2003; Niimura 2012; Niimura et al. 2014, 2018). The olfactory system operates through a combinatorial coding scheme where the relationship between odorants and ORs is not one-to-one but rather multiple-to-multiple. In other words, each odorant is recognized as a combination of activated ORs. This mechanism allows each species to detect a much larger number of odors than the actual count of OR genes encoded in the species’ genome. The OR gene family is known for containing numerous pseudogenes. For instance, in humans and African elephants, the number of OR pseudogenes exceeds that of functional genes, with ∼440 pseudogenes in humans and ∼2,200 in African elephants. Additionally, frequent gene gains and losses characterize this gene family (Niimura and Nei 2007). OR genes are expressed in the main olfactory epithelium (MOE) within the nasal cavity and were first identified in rats in 1991 (Buck and Axel 1991).

**Table 1:** Five multigene families of GPCRs for mammalian chemosensory systems

	OR	V1R	V2R	T1R	T2R
Expression	Olfactory epithelium	VNO		Taste buds	
Ligands	Odorants	Volatile pheromone	Peptide pheromone	Sweet/umami tastants	Bitter tastants
Exons	Single	Single	Multiple	Multiple	Single
Species	# of genes				
Human	398 (442)	5 (115)	0 (20)	3	24 (10)
Chimpanzee	397 (442)	4 (102)	0 (17)	3	25 (12)
Macaque	350 (340)	0 (60)	0 (11)	3	27 (14)
Marmoset	352 (236)	8 (55)	0 (8)	3	20 (10)
Mouse	1,130 (236)	239 (153)	121 (158)	3	36 (7)
Rat	1,207 (560)	108 (108)	79 (142)	3	36 (5)
Dog	811 (289)	9 (54)	0 (9)	3	14 (4)
Cow	1,186 (1,098)	40 (43)	0 (16)	3	17 (12)

The table includes the numbers of intact genes, along with the total of truncated genes and pseudogenes in parentheses. The numbers of OR genes are derived from Niimura et al. (2014, 2018), V2R and T1R genes are from Nei et al. (2008), V1R genes are from Nei et al. (2008) and Young et al. (2010), and T2R genes are from Hayakawa et al. (2014).

Most mammals possess a secondary olfactory organ known as the vomeronasal organ (VNO), situated between the nasal and oral cavities. Initially considered specialized for pheromone detection, the VNO is now believed to share some functions with the MOE (Liberles 2014). The VNO and MOE are distinctly separate at the molecular level. Sensory neurons in the apical and basal regions of the VNO express vomeronasal receptors type 1 and type 2 (V1Rs and V2Rs), responsible for detecting pheromones from volatile molecules like sulfated steroids and water-soluble peptides, respectively (Niimura et al. 2020). The number of both V1R and V2R genes varies significantly among mammalian species (Young and Trask 2007; Young et al. 2010). The absence of the VNO in adult hominoids (humans and apes) and Old World monkeys correlates with very few or no functional V1R/V2R genes in their genomes (Table 1). It has been suggested that the number of intact V1R genes relates to the morphological complexity of the VNO (Takami 2002; Grus et al. 2005). Functional V2R genes are sparsely distributed in the phylogeny of mammals. Currently, intact V2R genes have been observed exclusively in rodents, strepsirrhines (a primate suborder including lemurs and lorises), opossums, and the platypus (Young and Trask 2007; Dong et al. 2012; Hohenbrink et al. 2013). Notably, while dogs and cows possess functional VNO and V1R genes, they lack functional V2R genes.

Taste receptors are expressed in the taste buds of the tongue. Among the five basic tastes, sweet, umami, and bitter tastants are detected by two multigene families of GPCRs, known as taste receptors type 1 and 2 (T1Rs and T2Rs; Chandrashekar et al. 2006). Generally, mammalian species possess three T1R genes: T1R1, T1R2, and T1R3 (Table 1). T1Rs function as heterodimers; T1R2 + T1R3 and T1R1 + T1R3 form receptors for sweet and umami tastants, respectively. In contrast, the T2R genes form a relatively large multigene family with up to ∼40 member genes in mammals, indicating the importance of detecting bitter substances, often harmful to animals. Interestingly, T1R genes exhibit sequence similarities to V2R genes, while T2R genes share sequence similarities with V1R genes. Therefore, vomeronasal and taste receptor genes share a common evolutionary origin. V1R and T2R genes lack introns, resembling OR genes, whereas V2R and T1R genes are encoded by 6 to 7 exons and possess long N-terminal tails.

There has been a long-standing debate regarding how different senses interact with each other. It has been proposed that increased acuity in one modality of senses can lead to the decline of another. For instance, studies by Keesey et al. (2019) and Stöckl et al. (2016) illustrated a trade-off between vision and olfaction in *Drosophila* and Lepidopteran insects, respectively. This trade-off is likely due to the substantial energy costs needed to maintain neural systems for sensation within a limited energy budget (Niven and Laughlin 2008). In the case of mammals, a similar trade-off between vision and olfaction has been suggested in primates based on comparisons of brain structure (Barton et al. 1995; Barton and Harvey 2000). Gilad et al. (2004) argued that the loss of OR genes coincided with the acquisition of full trichromatic vision. They examined the fraction of pseudogenes among 100 randomly selected OR gene sequences from each of 19 primate species. However, subsequent studies by Matsui et al. (2010) and Niimura et al. (2018) revealed that the acquisition of full trichromatic vision and the loss of OR genes are not directly linked when examining the entire repertoires of OR genes identified from whole genome sequences. Instead, Niimura et al. (2018) demonstrated that the rate of OR gene losses accelerated during primate evolution in two specific instances: (i) at the ancestral branch of haplorhines, where significant changes occurred in eye and nose morphology, and (ii) at the ancestral branch of leaf-eating colobines, where the diet shifted from mainly consuming fruit (frugivory) to predominantly eating leaves (folivory).

Cetaceans, which include toothed whales (Odontoceti) and baleen whales (Mysticeti), present another instance of trade-off between different sensory modalities. These marine mammals have undergone a significant reduction in olfactory and taste receptor genes, possibly due to their transition from a terrestrial to an aquatic environment (Feng et al. 2014; Zhu et al. 2014; Kishida et al. 2015; Kishida 2021). Odontocetes have completely lost their olfactory nervous systems, opting instead for the development of an echolocation system. This system involves emitting clicking sounds and measuring the time lapse between the emitted sounds and their echoes to create three-dimensional images of surrounding objects. In contrast, mysticetes have a considerably reduced but fully functional olfactory system (Kishida 2021). As the olfactory systems degenerate, odontocetes have only 10 to 20 intact OR genes, while mysticetes possess a larger repertoire of intact OR genes (50 to 100) compared with odontocetes (Liu et al. 2019; Kishida 2021). The loss of chemosensation in cetacean evolution is reported to have occurred gradually through multiple steps (Kishida et al. 2015). Kishida and Thewissen (2012) proposed that the diminished chemosensation in odontocetes was not directly linked to the adoption of echolocation. However, a more recent study by Springer and Gatesy (2017) suggests a trade-off between the two sensory systems. It is worth noting that pinnipeds and sirenians, which have independently adapted to aquatic life apart from cetaceans, also exhibit reduced OR genes compared with their terrestrial relatives (Beichman et al. 2019; Liu et al. 2019).

Bats have also developed a sophisticated echolocation system independent of odontocetes. Studies by Hayden et al. (2010) and Hayden et al. (2014) found no evidence of a trade-off between the development of echolocation and the loss of olfaction when examining the OR gene repertoires of various bat species. However, there is a proposal that a sensory trade-off between vision and echolocation has occurred in bats at the genetic level (Zhao et al. 2009; Shen et al. 2013; Gutierrez et al. 2018; Wu et al. 2018).

While the association between different sensory modalities in evolutionary processes has garnered scientific attention as mentioned above, there has been relatively less exploration of evolutionary patterns among different modalities of chemical senses, olfaction, pheromone detection, and taste. Did a trade-off between the sense of smell and taste occur during evolution? Alternatively, if there is a development or decline in the sense of smell within a specific lineage, does this correspondingly influence the development or regression of the sense of taste? The objective of this study is to investigate the interplay among different chemical senses by comparing the evolutionary dynamics among families of chemosensory receptor genes responsible for detecting odors, pheromones, and tastants.

Table 1 highlights that in rodents, the quantities of V1R and V2R genes are notably higher compared with those in other orders, while the counts of OR and T2R genes are slightly larger or similar to those in other mammalian orders. The order Rodentia is categorized into either five suborders, Sciuromorpha, Castorimorpha, Myomorpha, Anomaluromorpha, and Hystricomorpha (Carleton and Musser 2005), or three suborders, Sciuromorpha, Supramyomorpha (which includes Castorimorpha, Myomorpha, and Anomaluromorpha), and Hystricomorpha (D’Elía et al. 2019). In both systems of classification, Hystricomorpha stands out as the most morphologically and ecologically diverse among these suborders (as described below). The array of chemosensory receptor genes present in each species' genome is influenced by its habitat. Consequently, Hystricomorpha is the most suitable group of species to explore the evolutionary dynamics of these gene families. For this reason, this study has specifically focused on Hystricomorpha.

Hystricomorph rodents are characterized as hystricomorphous, indicating an enlarged infraorbital foramen that allows the passage of the medial masseter muscle (Honeycutt 2009). Notable species within this category include porcupines, naked mole-rats, capybaras, and even domesticated animals like guinea pigs. These suborder members display significant diversity in their habitat, behavior, and physical characteristics. For instance, naked mole-rats, nearly devoid of hair, live in elaborate underground eusocial communities. Nutrias predominantly inhabit freshwater embankments, constructing nests with intricate tunnel systems with passages and chambers. Porcupines, for protection, erect sharp quills composed of modified hair. Furthermore, Hystricomorpha species vary considerably in size and weight, spanning from the tiny naked mole-rat (30 to 80 g) to the larger capybara (35 to 66 kg). Animals of the Hystricomorpha species predominantly consume a wide array of plant-based foods, including fruits, seeds, nuts, roots, bulbs, bark, shrubs, grass, and grains (Wilman et al. 2014). For instance, common gundis inhabiting rocky areas in North Africa are herbivores, exclusively consuming a variety of plants. Central American agoutis primarily feed on fruits and drupes, while grasscutters, also referred to as greater cane rats, mainly consume grass. These ecological and dietary distinctions might be partly explained by differences in their chemical senses.

In this study, we aimed to explore the relationship between various chemical sensory modalities. We identified the almost complete repertoires of 5 GPCR gene families associated with chemical senses—OR, V1R, V2R, T1R, and T2R genes—from genome assemblies of 17 Hystricomorpha species and examined their evolutionary dynamics. Among these families, OR, V1R, and T2R genes share common structural features, having seven alpha-helical transmembrane (TM) regions without additional domains, and their coding sequences (CDSs) are intronless and ∼1 kb long. Due to the relatively short CDSs, nearly complete gene repertoires could be identified in each genome. On the other hand, V2R and T1R genes are split into multiple exons, causing their CDSs to often appear truncated in available genome assemblies, making accurate retrieval of the full-length CDSs challenging. Consequently, we focused on a specific exon, “exon 3,” for these genes, rather than the entire CDSs, to accurately estimate the number of encoded genes in a genome (Francia et al. 2015; Niimura et al. 2021). Exon 3 of a V2R/T1R gene encodes the majority of a ligand-binding domain and spans ∼810 bp, a length comparable with OR/V1R/T2R genes. Furthermore, we conducted new sequencing of the whole genome of the grasscutter to enhance the reliability of our findings in identifying these genes. By categorizing these genes into orthologous gene groups (OGGs), we accurately estimated the occurrences of gene gains and losses for each gene family in the evolution of Hystricomorpha. Through these analyses, we discovered that the OR, V1R, V2R, and T2R gene families had expanded and contracted in synchrony during their evolutionary process.

## Results

### Hystricomorph Genome Analysis

We analyzed the genome sequences of 17 species from 14 families within the suborder Hystricomorpha and used the mouse (*Mus musculus*) as the outgroup (supplementary table S1, Supplementary Material online). To assess the quality of the genome assemblies, we initially conducted Benchmarking Universal Single-Copy Orthologs (BUSCO) (Manni et al. 2021) and Core Eukaryotic Genes Mapping Approach (CEGMA) (Parra et al. 2007) analyses for all genomes. As expected, the mouse genome assembly exhibited the highest quality, with 96.4% complete orthologous genes from the Glires database of OrthoDBv10 (BUSCO) and 96.6% complete core vertebrate genes (CVGs) (CEGMA; supplementary fig. S1 and supplementary tables S2 and S3, Supplementary Material online). In contrast, the currently available grasscutter genome in the GenBank database (ThrSwi_v1_BIUU) displayed the lowest quality, with <60% of complete orthologous genes (BUSCO) and <50% of CVGs (CEGMA).

In order to enhance the quality of our genome data, we conducted a complete genome sequencing of the grasscutter, resulting in a new genome assembly labeled as ThrSwi_NIG_v1. The quality of this newly assembled grasscutter genome significantly surpassed that of ThrSwi_v1_BIUU. According to the BUSCO analysis, we identified 94.1% of complete orthologs and 93.3% of complete single-copy orthologs from the Glires data set, with the latter figure surpassing that of the mouse (supplementary fig. S1, Supplementary Material online). In the CEGMA analysis, 95.3% of the CVGs were identified in this assembly (supplementary table S3, Supplementary Material online). The size of the ThrSwi_NIG_v1 assembly stood at 2.18 Gb, closely resembling the estimated grasscutter genome size of 2.2 Gb. In contrast, the prior assembly, ThrSwi_v1_BIUU, measured significantly larger at 2.66 Gb, exceeding the estimated size. Consistent with this observation, the number of orthologs per core gene in the CEGMA analyses notably reduced from 1.62 (ThrSwi_v1_BIUU) to 1.01 (ThrSwi_NIG_v1).

**Fig. 1.**
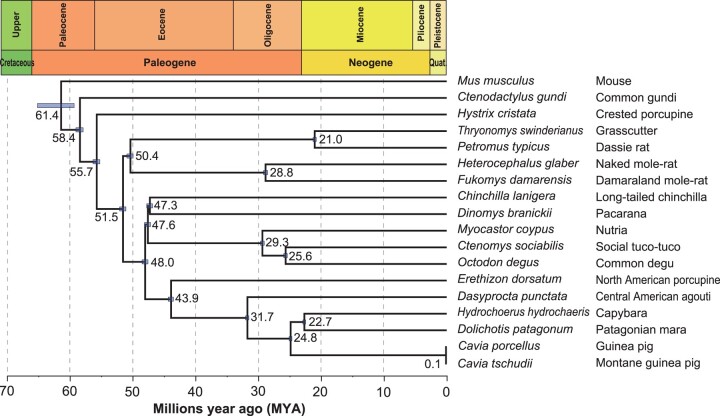
Phylogenetic tree of 17 Hystricomorpha species along with the mouse. The calibrated phylogenetic tree of Hystricomorpha with *M. musculus* as an outgroup was generated through Bayesian analysis in BEAST using a data set comprising 2,520 genes. Geological periods are depicted at the top. Each node displays the estimated divergence time (in MYA), with a bar representing the 95% credibility intervals for the respective node.

We proceeded by constructing a phylogenetic tree featuring the 17 Hystricomorpha species alongside the mouse as an outgroup. This was achieved using a concatenated sequence of 2,520 one-to-one orthologous genes, acquired through the BUSCO analysis (supplementary table S4, Supplementary Material online). Accurate phylogenetic relationships are crucial to estimate the occurrences of gene gains and losses in the evolution of Hystricomorpha (as detailed below). The tree topology generated via the maximum likelihood (ML) method closely resembled that of a prior study (Lacher et al. 2016), exhibiting 100% bootstrap support for all nodes (supplementary fig. S2, Supplementary Material online). Through divergence analysis, we were able to pinpoint the divergence among these rodents on an evolutionary time scale (Fig. 1).

### Chemosensory Receptor Genes in Hystricomorpha

**Fig. 2.**
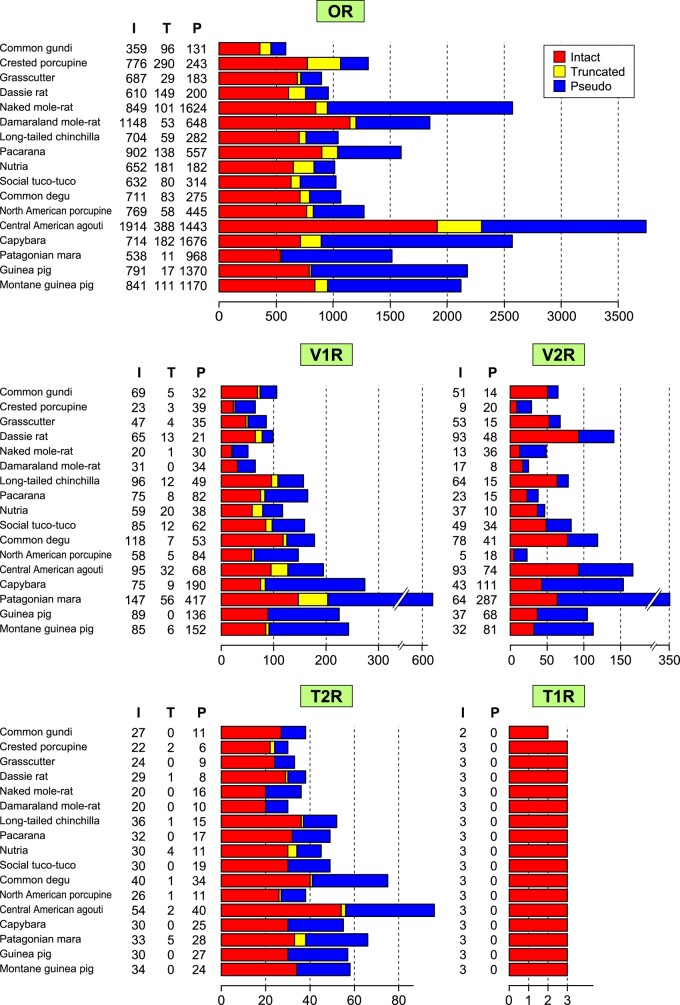
Number of OR, V1R, V2R, T1R, and T2R genes in 17 Hystricomorpha species. In the histograms for OR, V1R, and T2R genes, left, middle, and right bars indicate the number of intact genes (I), truncated genes (T), and pseudogenes (P), respectively, for each species. For V2R and T1R genes, the number of intact exon 3 sequences (I) and pseudogenes (P) is displayed.

To investigate the evolutionary patterns of chemosensory receptor gene families within Hystricomorpha, we identified OR, V1R, V2R, T1R, and T2R genes from the genome sequences of 17 Hystricomorpha species (Fig. 2; supplementary data S1 to S10, Supplementary Material online). For OR, V1R, and T2R genes, encoded by a single exon, we categorized the identified genes into three groups: intact genes, truncated genes, and pseudogenes. An intact gene is presumed to be functional, whereas a pseudogene contains disruptive elements such as stop codons, frameshifts, or gaps in conserved regions. A truncated gene represents a partially intact sequence found at the end of a contig, potentially becoming intact if the quality of the genome sequence is improved (Niimura and Nei 2007). Consequently, the number of intact genes depicted in Fig. 2 represents the lower bound of the number of functional genes encoded in each genome.

A V2R/T1R gene is typically comprised of 6 to 7 exons. Mouse V2R genes, with an average length of ∼25.3 kb (including introns), are more than 25 times longer than OR/V1R/T2R genes. Hence, the likelihood of a V2R/T1R gene being truncated in a genome assembly consisting of short contigs is substantially higher compared with an OR/V1R/T2R gene. Notably, the estimated probability of truncation for V2R genes is over 0.45 in 8 out of 17 genomes analyzed in this study (see Materials and Methods; supplementary table S1, Supplementary Material online). This suggests that for a significant portion of V2R genes from examined genome assemblies in this study, it is improbable to retrieve the entire CDSs.

Hence, instead of the entire CDSs, we focused on identifying the “exon 3” sequences of V2R/T1R genes (see Materials and Methods and Discussion for details). Consequently, we successfully pinpointed three intact exon 3 sequences of T1R genes in each species, excluding the common gundi, which lacks a T1R1 gene (Fig. 2; supplementary figs. S3 and S4, Supplementary Material online). We did not encounter any T1R pseudogene sequences. These findings demonstrate the stability in the number of T1R genes throughout hystricomorph evolution, rendering the T1R gene family less useful for exploring interactions among different gene families. Therefore, our subsequent analyses will focus on the remaining four gene families: OR, V1R, V2R, and T2R genes.

The number of intact genes varied across species for each of the four gene families. Across the 17 Hystricomorpha species, the mean numbers for intact OR, V1R, V2R, and T2R genes were 800, 73, 45, and 30, respectively (Table 2). The coefficients of variation, calculated by dividing the standard deviation by the mean, for intact OR, V1R, V2R, and T2R genes were 0.42, 0.45, 0.61, and 0.27, respectively. These figures indicate that the number of T2R genes displays less variability compared with the other gene families.

**Table 2:** Comparison of OR, V1R, V2R, and T2R gene families

	OR	V1R	V2R	T2R
# of intact genes in 17 Hystricomorpha	13,597	1,237	761	517
Mean # of genes per species	800	72.7	44.8	30.4
Coefficient of variation of # of genes per species	0.415	0.452	0.612	0.268
# of OGGs	654	33	8	21
Mean # of genes per OGG	20.8	37.5	95.1	24.6
Maximum # of genes per OGG	373	191	608	112
Standard deviation of # of genes per OGG	31.2	48.1	208	26.1
Rate of gene gain per gene	0.388	0.656	1.656	0.254
Rate of gene loss per gene	0.361	0.460	0.443	0.261

The numbers of intact genes for V1R and T2R exhibited a strong positive correlation (Spearman correlation coefficient *r*_S_ = 0.91; supplementary fig. S5, Supplementary Material online, top). Similarly, those for V1R and V2R genes (*r*_S_ = 0.64) and for V2R and T2R gene (*r*_S_ = 0.59) also demonstrated positive correlations. However, evolutionarily closely related species are expected to have similar gene counts due to shared ancestry. To address this, we employed a comparative method involving phylogenetically independent constants (Felsenstein 1985). Upon removing the phylogenetic influence, the observed correlations ceased (supplementary fig. S5, Supplementary Material online, bottom), except for a significant positive correlation between V1R and V2R genes, both of which participate in pheromone detection.

The majority of Hystricomorpha species possesses between 600 and 900 intact OR genes. Interestingly, Central American agoutis stand out with an exceptionally high count of intact OR genes (1,914), rivaling the numbers found in African elephants, which have the largest OR gene repertoire ever studied (Niimura et al. 2014). Conversely, the number of intact OR genes in common gundis was notably small (359).

Central American agoutis possess the largest collection of intact V2R genes (93) and T2R genes (54) as well. As far as our knowledge goes, this count of T2R genes is the highest ever documented among all mammalian species (Hayakawa et al. 2014; Li and Zhang 2014; Shang et al. 2017) (see Discussion). In contrast, naked mole-rats and Damaraland mole-rats exhibit the smallest T2R gene repertoires (20). Mole-rats possess unique ecological traits, dwelling underground and living in eusocial communities, seldom surfacing and primarily feeding on bulbs and tubers (Jarvis 1981; Jarvis and Bennett 1993; Oosthuizen et al. 2003; Buffenstein et al. 2022). Among the species surveyed, naked mole-rats also exhibit the fewest intact V1R genes (20).

Upon comparing the previous and newly sequenced grasscutter genome assemblies, it was evident that the number of truncated OR and V1R genes notably decreased, while the total number of genes did not significantly change (see supplementary fig. S6, Supplementary Material online). This outcome once more indicates a substantial improvement in the quality of the grasscutter genome assembly.

### Gains and Losses of Chemosensory Receptor Genes in the Hystricomorpha Evolution

In order to explore the evolutionary changes within the Hystricomorpha involving OR, V1R, V2R, and T2R genes, we identified OGGs for each gene family. An OGG represents a set of genes that originated from a single gene in the most recent common ancestor (MRCA) of the species under study. Consequently, we classified 13,597 OR genes, 1,237 V1R genes, 761 V2R genes, and 517 T2R genes identified from 17 Hystricomorpha species into 654, 33, 8, and 21 OGGs, respectively (Table 2; supplementary data S11 to S14, Supplementary Material online).

**Fig. 3.**
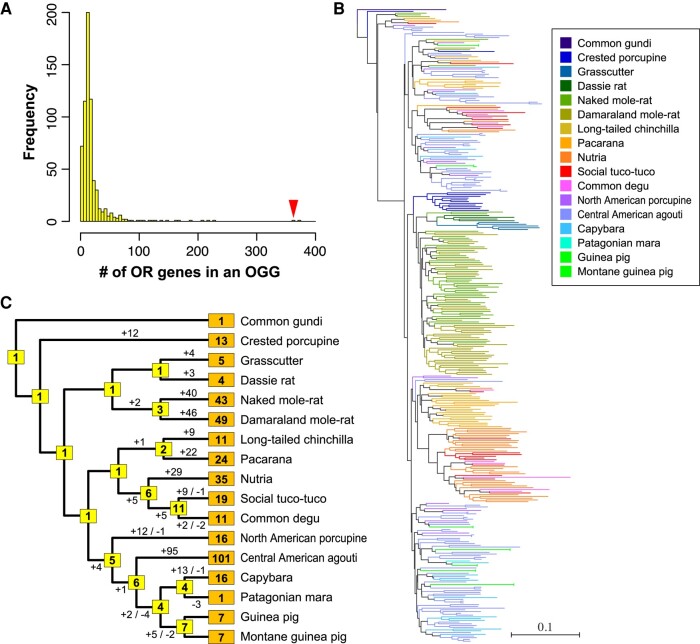
Expansion of OR genes within OGG2-27 in Hystricomorpha evolution. a) A histogram illustrating the number of OR genes belonging to each of the 654 OGGs. OGG2-27 is denoted by an arrowhead. b) A phylogenetic tree for 363 hystricomorph OR genes within OGG2-27. The color codes are shown on the right. The scale bar represents the number of amino acid substitutions per site. c) Depiction of gains and losses of OR genes within OGG2-27 in each branch of the Hystricomorpha evolution. The boxes on external and internal nodes represent the number of intact genes within OGG2-27 in each extant species and estimates of functional genes in the ancestral species, respectively.

The quantity of OR genes within each OGG exhibited significant variability among the OGGs (Fig. 3A), a pattern previously observed in placental mammals (Niimura et al. 2014). The ancestral OR gene of OGG2-27 in the MRCA of Hystricomorpha gave rise to 363 descendant genes across 17 Hystricomorpha species (Fig. 3B and C; supplementary data S11, Supplementary Material online). OGG2-27 is the most largely expanded OGG in the Central American agouti, with 101 OR genes from this species. Intriguingly, only five mouse OR genes (*Olfr1096* to *1100*) and three human OR genes (*OR8H1*, *OR8H2*, and *OR8H3*) belong to OGG2-27 (Niimura et al. 2014), indicating a specific expansion of OR genes within this OGG occurred in the hystricomorph lineage.

**Fig. 4.**
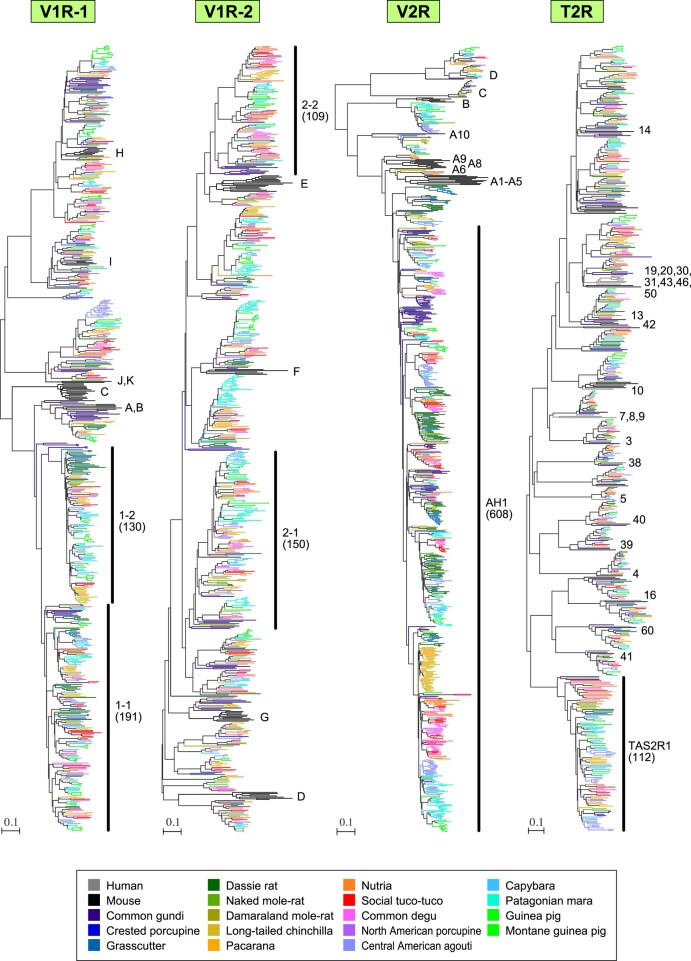
Phylogenetic trees for V1R, V2R, and T2R genes. The V1R genes were classified into two groups: V1R-1 containing clades A, B, C, H, I, J, and K and V1R-2 containing clades D, E, F, and G. V1R-1 and V1R-2 trees encompassed 613 and 624 V1R genes from 17 Hystricomorpha species, respectively, as well as 36 and 38 mouse V1R genes, respectively. The V2R tree was constructed using the exon 3 sequences of 761 hystricomorph V2R genes alongside 28 representative mouse genes. The T2R tree employed 517 hystricomorph T2R genes with 24 human and 36 mouse genes. The OGGs, which contained >100 genes from 17 Hystricomorpha species, are depicted by their OGG names along with the number of genes they include (in parentheses). For example, “1-1” in the V1R-1 tree denotes OGG-V1R-1-1, the largest OGG for V1R, housing 191 hystricomorph genes. In both V1R-1 and V1R-2 trees, the clade names for mouse genes align with those provided by Rodriguez et al. (2000) and Grus et al. (2005). In the V2R tree, subfamily names for mouse V2R genes are labeled according to Silvotti et al. (2011). In the T2R tree, gene names for human T2R genes are displayed (e.g. “14” represents *TAS2R14*). Color codes are outlined at the bottom, and the scale bar indicates the number of amino acid substitutions per site.

Figure 4 (V1R-1, V1R-2) illustrates the phylogenetic trees encompassing all 1,237 V1R genes detected among 17 Hystricomorpha species, alongside representative mouse V1R genes (supplementary figs. S7 to S10, Supplementary Material online). The V1R genes in rodents are divided into two distinct groups based on their sequence similarities: one comprises clades A, B, C, H, I, J, and K, and the other encompasses clades D, E, F, and G (Rodriguez et al. 2000; Grus et al. 2005). Therefore, the phylogenetic trees are presented separately for these two groups. The largest OGG for V1R, OGG-V1R-1-1, contains 191 genes from the 17 Hystricomorpha species (supplementary data S12, Supplementary Material online). Additionally, 3 more OGGs (OGG-V1R-1-2, OGG-V1R-2-1, OGG-V1R-2-2) included over 100 genes from these 17 species.

Figure 4 (V2R) displays the phylogenetic trees comprising all the exon 3 sequences of 761 V2R genes found in Hystricomorpha, along with representative mouse V2R genes (supplementary figs. S11 and S12, Supplementary Material online). Notably, there is a hystricomorph-specific OGG (OGG-V2R-AH1) that demonstrates extensive expansion, housing 608 V2R genes from the 17 Hystricomorpha species (supplementary data S13, Supplementary Material online). In stark contrast, OGG-V2R-C displays considerable evolutionary conservation, manifesting one-to-one orthologous relationships across all 17 Hystricomorpha species. This particular OGG is orthologous to the subfamily C of mouse V2R genes, which represents the most basal group and is coexpressed with V2R genes belonging to subfamilies A, B, and D (Silvotti et al. 2011; Brykczynska et al. 2013; Francia et al., 2015; Tirindelli 2021).

Figure 4 (T2R) shows the phylogenetic tree presenting all 517 T2R genes alongside human and mouse T2R genes (supplementary figs. S13 and S14, Supplementary Material online). Among the identified OGGs for T2R, the largest is OGG-TAS2R1, containing 112 genes, and it is the only OGG with over 100 genes (supplementary data S14, Supplementary Material online). Among the T2R genes of Central American agoutis, 24 belong to this OGG. This particular OGG is orthologous to human *TAS2R1* gene. Previous research also noted a specific expansion of *TAS2R1* orthologs in the hystricomorph lineage (Hayakawa et al. 2014).

The variation in the number of genes across each OGG was more pronounced for V1R and V2R compared with OR and T2R (Table 2, supplementary fig. S15, Supplementary Material online). The standard deviation of the number of V2R genes in each OGG (208) was notably larger than that of V1R (48.1; *P* < 10^−8^, *F*-test), OR (31.2; *P* < 10^−15^), and T2R genes (26.1; *P* < 10^−11^). Furthermore, the standard deviation of the number of V1R genes was significantly larger than that of OR (*P* < 10^−4^, *F*-test) and T2R genes (*P* = 0.006). These findings indicate that V1R and V2R genes experienced more dynamic evolutionary changes compared with OR and T2R genes. On the contrary, the number of T2R genes within each OGG exhibited considerably less variation, suggesting the relative stability of T2R genes during the course of Hystricomorpha evolution. Specifically, 12 out of the 21 OGGs included 14 to 17 T2R genes from the 17 Hystricomorpha species.

**Fig. 5.**
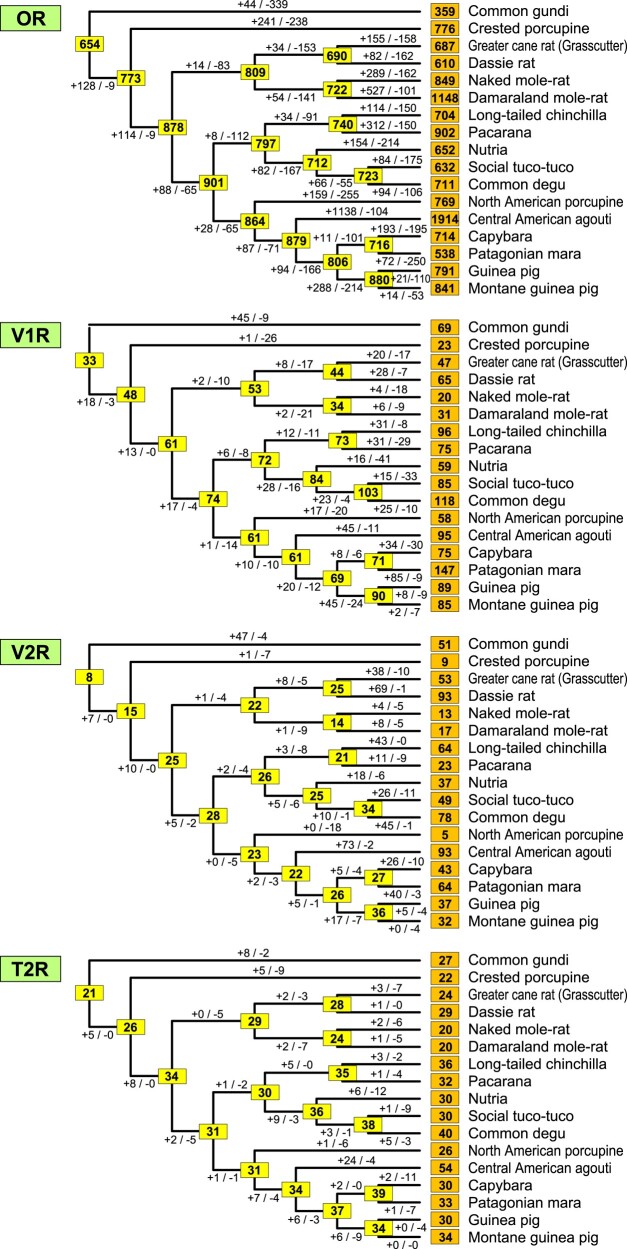
Gains and losses of OR, V1R, V2R, and T2R genes during Hystricomorpha evolution. The boxes on external and internal nodes represent the number of intact genes in each extant species and estimates of functional genes in the ancestral species, respectively. Additionally, it displays the estimated numbers of gene gains and losses in each branch of the Hystricomorpha phylogenetic tree.

Next, we estimated the number of gains and losses for OR, V1R, V2R, and T2R genes for each OGG in accordance with the Hystricomorpha phylogeny (Fig. 1), utilizing the reconciled tree method. The total count of gene gains and losses across all OGGs is illustrated in Fig. 5. In Fig. 5A, the chart shows a notably high frequency of gains and losses among OR genes during the course of evolution, as previously observed (Niimura and Nei 2007; Niimura et al. 2014). The findings also imply that although the number of OR genes varies considerably in extant Hystricomorpha species, those in the ancestral nodes appear to be relatively consistent (falling within the range of 700 to 900). Utilizing these figures, we computed the rates of gene gains/losses per gene during the evolution of Hystricomorpha for each gene family. The analysis revealed that both the rates of gene gains and losses were highest for V1R/V2R genes, followed by OR genes, with T2R genes exhibiting the lowest rates (Table 2).

**Fig. 6.**
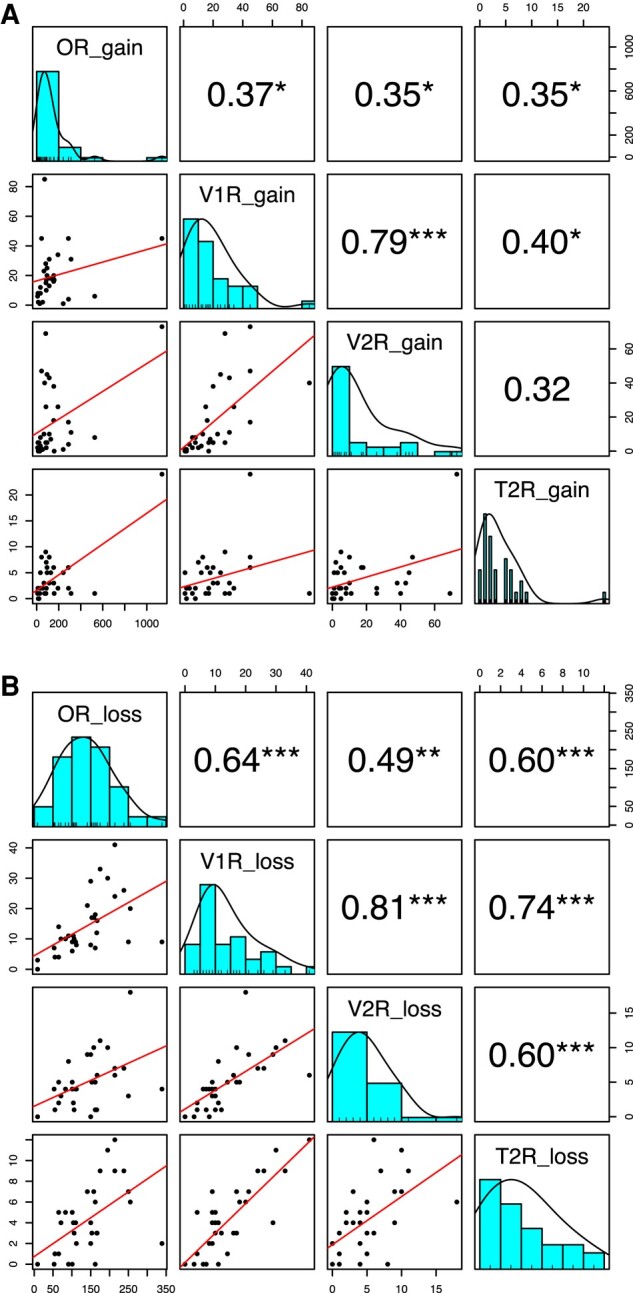
Correlation in the numbers of gene gains (a) and gene losses (b) per branch of the Hystricomorpha evolution among OR, V1R, V2R, and T2R genes. The histograms within squares on a diagonal line represent the distribution of the numbers of gene gains (a) or losses (b). The numbers within squares at the top right display Spearman's rank correlation coefficient. *P < 5%; **P < 1%; ***P < 0.1%. The graphs within squares at the bottom left indicate the scatterplot with a regression line.

We analyzed the correlation between the number of gene gains or losses along each branch of Hystricomorpha evolution among OR, V1R, V2R, and T2R genes (Fig. 6). The results demonstrated a significant positive correlation in the number of gains across these four gene families in most instances (*P* < 5%). Interestingly, there was a more stringent positive correlation in the number of losses (*P* < 0.1%). Particularly strong correlations were observed between V1R and V2R genes for both gene gains and losses, indicating notable associations (*P* < 0.1%).

## Discussion

In this study, we investigated the evolutionary dynamics among 4 chemosensory receptor genes—OR, V1R, V2R, and T2R—responsible for detecting odorants, pheromones, and tastants in 17 Hystricomorpha species. The findings reveal that (i) V1R/V2R genes exhibit the fastest gene turnover, followed by OR genes, while T2R genes display the slowest turnover. Additionally, (ii) gains or losses occurred synchronously among OR, V1R, V2R, and T2R genes during Hystricomorpha evolution. This synchronous fluctuation suggests that these four gene families expanded or contracted simultaneously throughout the evolutionary process, indicating no trade-off among different chemical sensing modalities.

Observation (i) can be elucidated by noting the functional distinctions among the four gene families. Ligands of V1Rs and V2Rs are species-specific pheromones. ORs are receptive to odorants that depend on distinct living environments in each species. On the other hand, T2Rs are attuned to detecting bitter taste compounds, a common signal of potential toxicity in most animals. Hence, it is plausible that V1R/V2R genes undergo the most rapid evolution, OR genes display intermediate evolutionary rates, and T2R genes are the most conserved across species. Similar contrasting evolutionary patterns of OR and V1R/V2R genes have been observed in a broader spectrum of vertebrate species (Grus and Zhang 2008). While previous studies have reported considerable variability in the number of T2R genes among mammals (Shi and Zhang 2006; Hayakawa et al. 2014; Li and Zhang 2014), our study revealed that the degree of variability is greater for OR, V1R, and V2R genes than for T2R genes, both across species and among OGGs (Table 2).

It should be noted that the current number of intact genes within a species is a result of both gene gains and losses over time. If an equal number of gains and losses occur, they counteract each other, resulting in no net change in the gene count. Since the frequency of gene gains and losses is extremely high for OR genes (Niimura and Nei 2007; Niimura et al. 2014), focusing on the numbers of gene gains and losses during evolution is more relevant than solely comparing the current gene numbers among existing species. Therefore, to explore the correlation between different gene families, we identified OGGs for each gene family and calculated the count of gene gains and losses separately for each branch in the Hystricomorpha phylogeny.

We should also note that the number of intact genes in each species cannot be considered as independent variables when examining the correlation between different gene families. This is due to the shared evolutionary history among any two species. Thus, removing the phylogenetic dependence is crucial to assess the significance of correlations, as demonstrated in supplementary fig. S5, Supplementary Material online (bottom). On the contrary, in the analyses presented in Fig. 6, such removal is unnecessary as the number of gene gains/losses occurring at each branch was assessed separately. Consequently, the number of gene gains/losses is considered an independent variable. In this study, the significance of the correlation among different gene families was detectable because we separately calculated the number of gene gains/losses in each branch using OGGs.

In this study, we focused on examining exon 3 sequences of V2R/T1R genes rather than analyzing the entire CDSs. The rationale for utilizing exon 3 sequences as a proxy of the entire CDS is in the following: (i) exon 3 predominantly encompasses the ligand-binding domain of a V2R. (ii) The phylogenetic analysis of exon 3 sequences exhibited a remarkably similar topology to that of full-length V2R genes (Francia et al. 2015; Niimura et al. 2021). (iii) Typically, exon 3 of a V2R/T1R gene encodes an average of ∼270 amino acids, a length comparable with that of an entire OR/V1R/T2R gene.

As a result, we found three intact exon 3 sequences of T1R genes and no pseudogenes in each species, with an exception of the common gundi which lacked the T1R1 gene responsible for the umami receptor (Fig. 2; supplementary figs. S3 and S4, Supplementary Material online). The fact that precisely three intact exon 3 sequences of T1R genes were identified in most of the examined species supports the notion that the exon 3 sequence can serve as a proxy for the full-length sequence. However, it is important to note that a single exon cannot fully represent the entirety of a gene with six or seven exons. Therefore, there is a limitation to this analysis.

We investigated the common gundi genome to detect the presence of the exons other than exon 3, but apart from an incomplete exon 6 sequence, we did not identify any of them. To examine whether the absence of the T1R1 gene in the common gundi genome is due to an incomplete genome assembly, we investigated the presence of adjacent genes (*Klhl21*, *Zbtb48*, and *Nol9*) to the T1R1 gene (supplementary fig. S16, Supplementary Material online). We found all these flanking genes in the genome sequences of all examined species, and they mostly shared the same scaffold as T1R1. However, in the common gundi, although all the flanking genes were on a single scaffold (PVKB01001179.1), the T1R1 gene itself was not identified (see supplementary information and supplementary tables S5 and S6, Supplementary Material online). Notably, there are no gaps in the genomic region containing these franking genes in the scaffold PVKB01001179.1. Therefore, it is possible that the T1R1 gene has undergone pseudogenization in the common gundi genome, although we cannot rule out the possibility of assembly errors. In this regard, it is noteworthy that certain mammals, such as carnivores, cetaceans, and bats, have lost one or more T1R genes in response to changes in diet, feeding habitats, or environmental conditions (Jiang et al. 2012; Zhao et al. 2012; Antinucci and Risso 2017; Wolsan and Sato 2022).

We found that the Central American agouti possesses a surprisingly large number of OR genes, comparable with that of the African elephant (Niimura et al. 2014). This observation does not appear to stem from an assembly error, because (i) the size of the Central American agouti's genome assembly stands at 3.01 Gb (supplementary table S1, Supplementary Material online), falling within the range of other Hystricomorpha species' genome sizes, and (ii) the amino acid sequence diversity among all identified OR genes in the Central American agouti closely aligns with that of the African elephant (supplementary fig. S17, Supplementary Material online). If the extensive presence of OR genes in the Central American agouti genome resulted from an erroneous assembly, it would imply an abundance of similar OR genes. Our analyses showed that 1,914 Central American agouti OR genes and 1,948 African elephant OR genes are divided into 1,160 and 1,080 groups, respectively, based on a 90% amino acid sequence identity threshold (supplementary fig. S17, Supplementary Material online). Thus, the OR genes in the Central American agouti exhibit comparable diversity with those in the African elephant, suggesting that the Central American agouti's genome does contain a large repertoire of OR genes. Interestingly, the Central American agouti also hosts the largest numbers of V2R (93) and T2R (54) genes among the examined Hystricomorpha species. This T2R count is even the highest among any mammals reported (Hayakawa et al. 2014; Li and Zhang 2014; Shang et al. 2017).

Why does the Central American agouti possess such extensive OR, V2R, and T2R gene repertoires? There are several reasons why this may be advantageous. Central American agoutis have a habit to bury nuts in the ground, storing them for later consumption (Smythe 1978). This behavior may require a heightened sense of smell and taste to locate hidden food sources and identify potential dangers. Additionally, Central American agoutis are frugivores, with ∼40% of their diet consisting of fruits (Wilman et al. 2014), making them crucial seed dispersers. In this regard, it is worth noting that frugivorous primates tend to have a larger number of OR genes compared with their nonfrugivorous relatives (Niimura et al. 2018). Moreover, Hayden et al. (2014) reported that specific subsets of OR genes (OR1/3/7 and OR2/13) have expanded in frugivorous bats. However, OGG2-27, the most largely expanded OGG in the Central American agouti, does not belong to either OR1/3/7 or OR2/13. Exploring whether the same OGGs expanded or contracted in parallel across two or more distinct lineages due to similar dietary shifts would be intriguing.

It is also possible that the extensive gene repertoires of the Central American agouti occurred neutrally, driven by “genomic drift” (Nei et al. 2008). This species' genome might be more tolerant toward gene duplication compared with other species. The analysis in Fig. 5 implies that the number of OR genes in the ancestral nodes of the Hystricomorpha phylogeny remains relatively stable across evolution, despite the significant variation in the current number of OR genes among extant species. This trend may indicate an optimal number of genes for Hystricomorpha species. In such a scenario, even if the number fluctuates neutrally due to random drift, it would eventually regress to this optimal value. Currently, the reason behind the presence of extensive OR, V2R, and T2R gene repertoires in the Central American agouti remains unclear. Performing comparative genomic analyses of its close relatives might offer further insights.

Among the examined species, Patagonian maras have the largest repertoires of intact V1R genes (147) and pseudogenes (417). This observation may be attributed to the unique mating system and social structure of this species. Unlike most mammals, Patagonian maras are monogamous, forming strong, lifelong pair bonds (Kessler et al. 2009). The male follows and guards the female wherever she goes, marking her as his territory by urinating directly on her.

In this research, we assembled a high-quality genome of the grasscutter, achieving over 90% completeness based on the expected BUSCO genes. Despite previous studies attempting to estimate the divergence time among distinct Hystricomorpha species, a consensus remains elusive. Our research estimated the divergence between Hystricomorpha and Myomorpha to be around 61.4 million years ago (MYA). This estimation aligns closely with some existing studies (Hallstrom and Janke 2010; Jameson et al. 2011; dos Reis et al. 2012; Upham and Patterson 2012; Ge et al. 2013; Pozzi et al. 2014; Shao et al. 2015; Laurin et al. 2022) but significantly differs from others (16 MYA in Ge et al. (2013) and 76.5 MYA in Fabre et al. (2012)). Additionally, the divergence estimate between guinea pigs and Montane guinea pigs was determined to be 0.107 MYA, consistent with Upham and Patterson (2012) but divergent from various other studies (Fritz et al. 2009; Fabre et al. 2012; Upham and Patterson 2015; Álvarez et al. 2017). Differences in these estimates are often related to the quantity and type of genes (nuclear or mitochondrial) employed. Notably, our study utilized the largest number of nuclear genes to date, thus yielding more accurate estimates of divergence times.

Hystricomorph rodents have a wide geographic distribution, populating every continent except Antarctica. The species belonging to Hystricomorpha exhibit substantial diversity in body size, ecological niches, and morphological traits. Exploring the genes responsible for chemosensory receptors in these species could offer insights into the relationship between genes and the variation in these traits. In this research, we have produced an updated version of the grasscutter genome (ThrSwi_NIG_v1). The grasscutter, found in sub-Saharan Africa, weighs between 3.5 and 4.5 kg and is a common source of meat consumed by humans, particularly in Western Africa (Adenyo et al. 2017; Dery et al. 2020). The molecular data obtained on chemosensory receptor genes, along with the whole genome sequence, will be instrumental in conducting further genetic analyses of grasscutters and other species within the hystricomorph group.

In summary, our analyses showed that the four gene families involved in the chemical senses (OR, V1R, V2R, and T2R genes) expanded or contracted synchronously during the evolution of Hystricomorpha. This means that when the number of genes in one family increased or decreased, the others tended to do the same. In other words, there was not a trade-off between different chemical senses. This coherence might be explained by differences in genome tolerance to gene duplications across species, as mentioned earlier. However, the precise evolutionary mechanisms responsible for this synchronicity across the gene families related to chemosensory receptors remain unclear, and further studies are needed to gain a better understanding of this phenomenon.

## Materials and Methods

### Evaluation of the Genome Assemblies

The genome assemblies of 17 Hystricomorpha species and mice were downloaded from the National Center for Biotechnology Information website (https://www.ncbi.nlm.nih.gov/assembly) (supplementary table S1, Supplementary Material online). The completeness of the genome assemblies was evaluated using BUSCO v5.0 (Manni et al. 2021) and CEGMA v2.5 (Parra et al. 2007). For the BUSCO analysis, the Glires data set from OrthoDBv10 (creation date: 2020 August 5) (Kriventseva et al. 2019) was used because it is the closest available lineage database to Hystricomorpha. BUSCO v5.0 uses MetaEuk (Levy Karin et al. 2020) and HMMER 3.1+ (Eddy 2011) to verify assembly completeness and gene prediction. The rest of the settings were default in the BUSCO analysis. For the CEGMA analysis, 233 CVGs that are shared as one-to-one orthologs by all vertebrate genomes were used (Hara et al. 2015).

### Grasscutter Genome Analysis

Genomic DNA from a male grasscutter was extracted from the muscle tissue using the following methods. Muscle tissue was lysed in lysis buffer (KURABO Genomic DNA Extraction Solution, KURABO Industries Ltd., Osaka, Japan) supplemented with proteinase K at a final concentration of 0.2 mg/mL. The tissue was lysed at 55°C for overnight, followed by an RNase A treatment for 1 h at 37°C at 0.15 ng/mL concentration. Genomic DNA was extracted using phenol/chloroform followed by chloroform. Genomic DNA was precipitated with isopropanol, washed with 70% ethanol, and dissolved in TE buffer.

For the whole genomic sequencing, one paired-end library, PE600, with 638 bp insert sizes on average, and four mate-pair libraries with different insert sizes (MP3000, 3,147 bp; MP6000, 6,157 bp; MP10000, 9,575 bp; MP15000, 13,457 bp) were generated from the genomic DNA. The five libraries were sequenced on an Illumina HiSeq 2500 platform. The grasscutter genome size was estimated as 2.2 Gb using k-mer distribution analysis in GenomeScope 1.0 (Vurture et al. 2017). The statistics of the new grasscutter genome assembly (ThrSwi_NIG_v1) were calculated using QUAST (Gurevich et al. 2013) with a sequence cutoff of 1,000 bp: The assembly was 2.18 Gb long with 20,779 scaffolds, a scaffold N50 length of 20,890,746, and a GC content of 42.5%. Raw sequencing reads and assembled genomes are available in the DDBJ/GenBank/EMBL with BioProject accession number PRJDB10995. Moreover, we developed a web browser for the new grasscutter genome to ensure that the genomic information can be more widely and effectively used by many researchers (https://grasscutter.nig.ac.jp/).

### ML Phylogenetic Analysis

The amino acid output of BUSCO analysis, which uses orthologous gene sequences in its database, was used for phylogenetic analysis. We selected the amino acid sequences of genes that were complete, single-copy, and present in all species examined in our study, identifying 2,520 sequences that met this criterion (supplementary table S4, Supplementary Material online). For each protein, we performed a local alignment using MAFFT v7.490 (Katoh and Standley 2013) with the “--maxiterate 1,000 --localpair” command. Low-quality alignments were filtered out using trimAI (Capella-Gutierrez et al. 2009) with the “-automated1” command. After alignment and quality trimming, we concatenated all sequences together. The resultant aligned FASTA file was then used for ML tree construction in raxmlGUI 2.0 (Edler et al. 2021). To determine the appropriate model, we used ModelTest-NG (Darriba et al. 2020) and found that JTT + I + G4 + F was the best fit. We then generated a RAxML tree with 1,000 bootstraps (Stamatakis 2014). The resulting tree was visualized and edited using FigTree (http://tree.bio.ed.ac.uk/software/figtree/).

### Divergence Dating

This analysis used aligned and quality-trimmed protein sequences obtained from BUSCO. The best fit evolutionary model for each protein sequence was selected using PartitionFinder2 (Lanfear et al. 2017) with “rclusterf” search strategy (Lanfear et al. 2014) and “--raxml” (Stamatakis 2014) command. PartitionFinder2 produced 14 different models for the protein sequences (supplementary table S4, Supplementary Material online). AMAS was used to concatenate proteins with similar evolutionary models into an amino acid supermatrix (Borowiec 2016). Divergence dating was performed using BEAST 2.6 (Bouckaert et al. 2019) with each amino acid supermatrix treated as a partition (14 partitions in total). All site models were unlinked, while clock models and trees were linked between the partitions. Each partition was assigned a different model based on the model predicted by PartitionFinder2. The relaxed log-normal was used in the clock model (Drummond et al. 2006), and different fossil calibrations were used to constrain the trees (supplementary information, Supplementary Material online). The Birth–Death model was chosen prior to tree generation, setting the MCMC chain length at 20,000,000 generations, sampling every 5,000 generations. Analyses were checked for convergence using Tracer v.1.7.1 (Rambaut et al. 2018). Trees before convergence were discarded as burn-in, and a single consensus tree was generated using TreeAnnotator. The final tree was generated using R package strap (Bell and Lloyd 2015). Posterior probabilities were added to the tree using FigTree (http://tree.bio.ed.ac.uk/software/figtree/). Our findings were also verified using resources from timetree.org (Kumar et al. 2017).

### Identification of OR Genes

OR genes were identified in the genome sequences of the 17 Hystricomorpha species (supplementary table S1, Supplementary Material online). We identified intact genes, truncated genes, and pseudogenes of ORs using the method described in Niimura (2013) with a modification described in the supplementary note in Niimura et al. (2018). To refine the OR genes identified above, these genes were further filtered using four additional steps (supplementary fig. S18, Supplementary Material online). (i) Genes encoded in contigs shorter than 1 kb were discarded. (ii) When a contig was embedded within another longer contig with a >99% nucleotide sequence identity and an OR gene encoded in one contig is >99% identical in amino acid sequence to the OR gene encoded in the other contig, the OR gene encoded in a shorter contig was discarded (Niimura et al. 2018). (iii) Intact/truncated OR genes with 100% amino acid sequence identity with another intact/truncated OR gene in the same species were discarded in the following way. When two intact OR genes were 100% identical in amino acid sequences without any gaps, either of the genes was discarded. When a truncated gene was a part of an intact gene with 100% identity in the amino acid sequence without any gaps, the truncated gene was discarded. When a truncated gene was a part of another truncated gene with 100% identity in the amino acid sequence without any gaps, the shorter truncated gene was discarded. (iv) When there were two truncated genes, one of which was missing the N-terminal end and the other was missing the C-terminal, and the amino acid sequence in an overlapped region was 100% identical to each other without any gaps, then the two truncated genes were combined into one sequence and regarded as an intact gene.

### Identification of V1R and T2R Genes

V1R and T2R genes were identified using a method similar to that used for OR genes, with slight modifications. V1R and T2R genes show weak similarity to each other; therefore, we identified V1R and T2R genes together. First, we performed TBLASTN searches (Altschul et al. 1997) with an e-value of 1e^−20^ against the whole genome sequences of the 17 Hystricomorpha species using the amino acid sequences of 318 V1R genes, 8 ancV1R genes, and 158 T2Rs genes as queries. Query sequences were obtained as follows: For V1R genes, we extracted functional V1R genes from 10 mammalian species (human, mouse lemur, mouse, rat, guinea pig, dog, cat, cow, horse, and elephant) from the supplementary data of Young et al. (2010). To eliminate highly similar sequences in the same species, we calculated the amino acid sequence identities between all possible pairs of V1R genes in each species and classified them into groups with a threshold of 80% amino acid sequence identity. We then extracted representative genes from each group to obtain a total of 318 (5 human, 21 mouse lemur, 76 mouse, 67 rat, 47 guinea pig, 7 dog, 16 cat, 25 cow, 31 horse, and 23 elephant) V1R genes that were used as queries. The amino acid sequences of eight ancV1R genes from eight mammalian species (mouse, rat, guinea pig, dog, cat, cow, horse, and elephant) were obtained from Zhang and Nikaido (2020). The amino acid sequences of 24 human, 36 mouse, 36 rat, and 31 guinea pig T2R genes were obtained from Hayakawa et al. (2014). Additionally, 14 dog and 17 cow T2R genes downloaded from GenBank were also used as queries.

Because the query sequences are similar to one another, multiple query sequences may hit the same genomic region. We therefore extracted the “best-hit” sequence for each genomic region, which corresponds to a query showing the lowest e-value among all queries that hit a given genomic region (Niimura 2013). The best-hit sequences were classified into three categories according to the query: V1R, ancV1R, and T2R. To extract intact genes from the best-hits, the following filtering process was conducted: First, the best-hit sequences shorter than 250 amino acids were discarded. We then extended each of the remaining best-hit sequences along the DNA to extract the longest CDS, starting from the initiation codon and ending with the stop codon. If the extracted CDS was shorter than 250 amino acids, the sequence was discarded.

Subsequently, the criteria for selecting intact sequences among the candidate sequences obtained above differed between V1R and T2R genes. The methods used for the V1R genes are as follows. Because we extracted the longest CDSs using the criteria above, some sequences contained excessively longer N-terminal sequences than the known V1R genes. Therefore, we chose the most appropriate ATG codon as an initiation codon among the multiple ATG codons located in the N-terminal portion in the following manner. We constructed a multiple alignment using all remaining sequences from each species together with 76 mouse V1R genes, which were used as queries in the TBLASTN searches mentioned above. The 24th amino acid of mouse Vmn1r1 (GenBank accession number, NP_001160200.1) is G (glycine), which is relatively well conserved among all mouse V1Rs. Therefore, we designated this amino acid position in each sequence as “0” (origin). We then counted the number of amino acids before the amino acid position 0 for each sequence. We selected an ATG codon located at the most appropriate position among the multiple ATG codons as an initiation codon in the following manner. (i) If the length of the N-terminal portion was zero, the sequences were discarded. (ii) Otherwise, we chose the ATG codon that is located at the most upstream position among all ATG codons existing between position −40 and position 0. Next, we constructed multiple alignments of the remaining sequences using MAFFT (Katoh and Standley 2013) and visually inspected them. Sequences shorter than 280 amino acids were excluded. Next, we predicted the TM helical regions for each of the 76 query genes using TMHMM-2.0 (Krogh et al. 2001). The 15-amino-acid region from position 274 to position 288 (“MFVSSGYATFSPLVF”) of Vmn1r1 corresponds to the 7th TM region for most of the 76 queries. Here, we call this region as the “7TM region.” If the number of gaps within the 7TM region in a multiple alignment was seven or more, such sequences were eliminated. All remaining sequences were regarded as intact V1R genes because they did not contain any gaps in well-conserved regions.

Intact T2R genes were selected from the candidate sequences in a manner similar to that used for V1R genes. We chose the most appropriate ATG codon as an initiation codon among the multiple ATG codons located in the N-terminal portion in the following manner. We constructed a multiple alignment using all remaining sequences from each species, together with 24 human and 36 mouse T2R genes used as queries in the TBLASTN searches. The 16th amino acid of human TAS2R5 (GenBank accession number, NP_061853.1) is E (glutamic acid), which is relatively well conserved among all human and mouse T2Rs. Moreover, for most human and mouse T2Rs, the length of the N-terminal portion prior to this amino acid is 15. We designated this amino acid position in each sequence as “0” (origin) and counted the number of amino acids before the amino acid position 0 for each sequence. We selected ATG as the initiation codon in the following manner. (i) If the length of the N-terminal portion was zero, the sequences were discarded. (ii) Otherwise, we chose the ATG codon that is located at the most upstream position among all ATG codons existing between position −27 and position 0. Finally, we constructed multiple alignments of the remaining sequences using MAFFT and visually inspected the alignment. We eliminated sequences shorter than 280 amino acids, and all remaining sequences were regarded as intact T2R genes because they did not contain any gaps in well-conserved regions.

Next, we identified truncated genes from the nonintact best-hit sequences. The criteria used to identify V1R and T2R truncated genes were the same as those for OR genes. All the best-hit sequences for V1R and T2R gene queries, excluding intact and truncated genes, were regarded as V1R and T2R pseudogenes, respectively.

Finally, the intact genes, truncated genes, and pseudogenes of V1R and T2R identified above were further refined by the additional four steps, which were used for the filtering processes for OR genes, as described above.

### Estimation of the Probability of Truncation of V2R Genes

We first calculated the mean length of 121 functional V2R genes in mice obtained in the “Identification of Exon 3 Sequences of V2R and T1R Genes” section. The coordinates of CDSs of these genes were retrieved from the file “Mus_musculus.GRCm39.104” downloaded from the Ensembl website (https://asia.ensembl.org/). The length of each V2R gene was calculated as a distance between the positions of the initiation codon and the stop codon. The mean length *L* of the V2R genes was calculated to be 25.3 kb.

Using *L*, the probability of truncation of a V2R gene for a given genome assembly was estimated in the following way. Suppose that the genome assembly is composed of *n*_1_ scaffolds that is longer than *L* and *n*_2_ scaffold that is shorter than or equal to *L*. Let us consider the situation that a V2R gene is located on a scaffold longer than *L* (top, supplementary fig. S19, Supplementary Material online). In this case, when the center of a V2R gene is located within the region of *L*/2 at either end of the scaffold, the gene is truncated, and the entire CDS cannot be retrieved. On the other hand, if a V2R gene is located on a scaffold shorter than or equal to *L*, the gene is truncated regardless of a location of the center of the gene (bottom, supplementary fig. S19, Supplementary Material online). Therefore, the probability *P* of truncation of a V2R gene can be estimated as


$$P=\left(n_{1}L+\overset{n_{1}+n_{2}}{\underset{i=n_{1}+1}{\sum }}\;S_{i}\right)/N$$

where *S_i_* is the length of *i*-th longest scaffold in the genome assembly and *N* is the entire length of the genome assembly.

### Identification of Exon 3 Sequences of V2R and T1R Genes

We first downloaded Vmn2r1 paralogs in mice from the Ensembl website (https://asia.ensembl.org/), which included V2R genes, T1R genes, and non-V2R/T1R GPCR genes such as the calcium-sensing receptor (CaSR) gene. We then constructed a multiple alignment of the amino acid sequences of these genes using “Mus_musculus.GRCm39.pep.all” file by MAFFT (Katoh and Standley 2013) and extracted the exon sequences that correspond to the exon 3 of Vmn2r1 by visual inspection. We call these sequences “exon 3” regardless of the difference in exon/intron structures, though for some genes, the corresponding exon is not actually the third exon. As a result, we obtained 128 unique exon 3 sequences of 121 V2R, 3 T1R, and 4 non-V2R/T1R GPCR genes. These sequences were used as queries for TBALSTN searches (Altschul et al. 1997) against the 17 Hystricomorpha genome assemblies with an e-value of 1e^−20^. We then extracted “best-hit” genomic regions to any of the 128 query sequences as explained in “Identification of V1R and T2R Genes” section. When the translated amino acid sequence for a best-hit DNA sequence were shorter than 250 amino acid long or contained an interrupting stop codon or a frameshift, the sequence was discarded. Next, we constructed a phylogenetic tree using the translated amino acid sequences of the remaining sequences together with the 128 query sequences and identified V2R and T1R genes according to the tree topology. Hystricomorpha V2R genes identified here were further classified into seven V2R gene clades, AH, A1-5, A8, A10, B, C, and D. The clade names of Hystricomorpha V2R genes were according to the nomenclature of mouse V2R genes (Silvotti et al. 2011) which formed a monophyletic clade with these genes, with the exception of AH clade containing only Hystricomorpha genes.

Next, we identified putative exon–intron boundaries that meet the “GT-AG rule,” which states that the first two and the last two nucleotides of introns are GT and AG, respectively, for each sequence. We did not consider noncanonical splice sites for 2 reasons: (i) all 121 exon 3 sequences of mouse V2R genes adhere to the GT-AG rule, and (ii) the overwhelming majority (>98.7%) of splice sites in mammalian genes is reported to follow the GT-AG rule (Burset et al. 2000). We constructed a multiple alignment for each clade separately with mouse query sequences. Each of the sequence was elongated to find putative exon–intron boundaries that met the GT-AG rule within a 15-nucleotide region from the end of the best-hit sequence. If there were two or more positions that met the GT-AG rule at either end of the sequence, the most appropriate position was adopted by visual inspection. The sequence lacking an appropriate exon–intron boundary meeting the GT-AG rule at either end was discarded, and the remaining sequences were regarded as intact sequences of the exon 3 of V2R genes. Exon 3 sequences of T1R genes were identified in a similar manner. After excluding intact V2R sequences, the sequences that best-hit to the mouse V2R queries with an e-value less than 1e^−20^ and that resided in contigs not shorter than 1 kb were regarded to be V2R pseudogenes.

### Construction of Neighbor-Joining Phylogenetic Trees

A neighbor-joining (NJ) tree (Saitou and Nei 1987) was constructed with Poisson correction (PC) distance using the LINTREE program (Takezaki et al. 1995). Multiple alignments of translated amino acid sequences were generated by the program MAFFT (Katoh and Standley 2013).

### Construction of ML Phylogenetic Trees

Amino acid sequences of V1R-1, V1R-2, and T2R genes and exon 3 sequences of V2R and T1R genes were aligned separately using the Clustal Omega (v.1.2.4) (Sievers et al. 2011) with amino acid setting (--seqtype=Protein). V1R-1 and V1R-2 genes were aligned independently. The phylogenetic trees were constructed by IQ-TREE2 (v.2.0.7) (Minh et al. 2020) based on ML method with amino acid setting (--seqtype AA). We executed 500 nonparametric bootstrap replicates. The best-fit substitution models for constructing phylogenetic tree were estimated by ModelFinder (Kalyaanamoorthy et al. 2017) implemented in IQ-TREE2 with the option considering standard model selection (-m TESTONLY --msub nuclear --seqtype AA). The optimal substitution model for each alignment was selected based on the value of Bayesian information criterion (BIC): JTT + F + I + G4 (V1R-1), JTT + F + G4 (V1R-2), JTT + F + G4 (T2R), JTT + G4 (V2R), and JTT + G4 (T1R). The obtained phylogenetic tree was visualized using FigTree (v.1.4.4).

### Identification of OGGs among Hystricomorph OR Genes

Intact OR genes identified from the genomes of 17 Hystricomorpha species were classified into OGGs among Hystricomorpha (hystricomorph-OGGs). For this purpose, we used 781 OGGs among placental mammals (placental-OGGs) identified in Niimura et al. (2014). If gene duplication(s) occurs in the branch between the MRCA of placental mammals and that of Hystricomorpha, one placental-OGG corresponds to two or more hystricomorph-OGGs. Therefore, the basic strategy is to assign each hystricomorph OR gene to one of the placental-OGGs and then divide placental-OGGs into hystricomorph-OGGs.

In the process of OR gene identification, we used 781 consensus sequences, each of which was constructed from a multiple alignment of all intact OR genes contained in each of the 781 placental-OGGs, as queries of TBLASTN searches (Niimura et al. 2018) (see supplementary fig. S18, Supplementary Material online). Each hystricomorph intact OR gene “best-hit” to one of the 781 queries (Niimura 2013). Based on these facts, a given hystricomorph intact OR gene was assigned to the placental-OGG from which its best-hit query was generated.

Next, we constructed an NJ phylogenetic tree for each placental-OGG separately using all hystricomorph intact OR genes assigned to the OGG and all intact OR genes from 13 placental mammals belonging to the OGG identified in Niimura et al. (2014). We also constructed a phylogenetic tree for placental-OGG using all hystricomorph OR genes in the OGG together with the intact OR genes only from mice and rats, rather than the 13 species. We then examined whether the hystricomorph OR genes were monophyletic or paraphyletic in each phylogenetic tree. When all hystricomorph OR genes assigned to a given placental-OGG formed a monophyletic clade, the hystricomorph OR genes were regarded as members of the candidate hystricomorph-OGG. Phylogenetic trees showing paraphyly of hystricomorph genes were further examined. From each of the Hystricomorpha-paraphyletic trees, we extracted phylogenetic clades that met either of the following conditions: (i) a clade that was supported with a >70% bootstrap value and contained a part (not all) of hystricomorph OR genes assigned to the OGG and at least one nonhystricomorph genes or (ii) a clade with a >70% bootstrap value that contains a part (not all) of hystricomorph OR genes assigned to the OGG, and the tree topology suggests that the divergence of the clade occurred before the separation of Hystricomorpha and non-Hystricomorpha with a >70% bootstrap support. The hystricomorph genes included in such a clade were regarded as members of the candidate hystricomorph-OGG. For each placental-OGG, the genes included in such a clade were excluded, and a phylogenetic clade was constructed again. This process was iteratively performed until no such clades were extracted and the remaining hystricomorph genes were regarded as members of another candidate hystricomorph-OGG.

Finally, for each of the candidate hystricomorph-OGGs, an NJ phylogenetic tree was constructed using only hystricomorph OR genes assigned to the OGG. For each phylogenetic tree, we examined whether OR genes from the common gundi, the most basal species examined in the Hystricomorpha phylogeny, showed monophyly or paraphyly in each phylogenetic tree. When a tree consisted of two clades, each of which contained both gundi and nongundi genes, and the separation of the two clades was supported with a >70% bootstrap value, the candidate hystricomorph-OGG was subdivided into two hystricomorph-OGGs. All constructed phylogenetic trees were visually inspected to confirm that the hystricomorph-OGGs were appropriately identified.

### Identification of OGGs among Hystricomorph V1R, V2R, and T2R Genes

We classified 1,237 intact V1R genes identified from 17 Hystricomorpha species into OGGs among Hystricomorpha. We first constructed an NJ phylogenetic tree using all 1,237 V1R genes and 74 representative mouse V1R genes from Young et al. (2010) that were used as queries for TBLASTN searches. We found that these V1R genes were clearly classified into 2 groups: one containing 649 genes that corresponds to clades A, B, C, H, I, J, and K and the other containing 662 genes that corresponds to clades D, E, F, and G, according to the nomenclature of Rodriguez et al. (2000) and Grus et al. (2005). We then constructed a phylogenetic tree for each of the two groups separately and subdivided the genes included in a given tree into smaller groups at the node supported with a high (>90%) bootstrap value. We iteratively performed these processes and identified OGGs among Hystricomorpha by visual inspection in the same criteria as those for OR genes.

For T2R genes, we first constructed an NJ phylogenetic tree using all 517 intact T2R genes identified from 17 Hystricomorpha species together with 24 human and 36 mouse T2R genes from Hayakawa et al. (2014) that were used as queries for TBLASTN searches. Most hystricomorph genes form monophyletic clades with some of the human and/or mouse T2R genes with >95% bootstrap values, showing clear orthologous relationships to human/mouse T2R genes. Hystricomorph genes that did not show clear orthology to human/mouse T2R genes were extracted, and a phylogenetic tree was constructed using these genes only. By visual inspection of the tree, OGGs among Hystricomorpha were determined using the same criteria as those for the OR genes.

Exon 3 sequences of V2R genes were also classified into OGGs. Each of the clades A1-5, A8, A10, B, C, and D corresponds to an OGG, while hystricomorph-specific AH clade was divided into two OGGs according to the criteria used for the identification of OGGs of OR genes.

### Estimation of the Numbers of Gene Gains and Losses

The number of gene gains and losses in each branch of Hystricomorpha phylogeny was estimated using the reconciled tree method with a 70% bootstrap value for the threshold of reconciliation (Niimura and Nei 2007). We calculated the numbers for each OGG separately, without using any outgroup sequences, as described in Niimura et al. (2014). The results for all the OGGs were compiled to generate Fig. 6.

The rate of gene gain per gene and that of gene loss per gene was calculated as follows: For each branch, the rate of gene gain/loss was calculated by dividing the number of gene gains/losses at the branch by the number of genes in the ancestral node to which the branch connects. The means were calculated among the values for all branches in the Hystricomorpha phylogeny for gene gains and losses separately.

## Supplementary Material

msae071_Supplementary_Data

## Data Availability

Raw sequencing reads and assembled genomes of the grasscutter (ThrSwi_NIG_v1) are available in the DDBJ/GenBank/EMBL with BioProject accession number PRJDB10995. Amino acid and nucleotide sequences for OR, V1R, V2R, T1R, and T2R genes identified in this study are available in supplementary data S1 to S10, Supplementary Material online.

## References

[msae071-B1] Adenyo C, Ogden R, Kayang B, Onuma M, Nakajima N, Inoue-Murayama M. Genome-wide DNA markers to support genetic management for domestication and commercial production in a large rodent, the Ghanaian grasscutter (*Thryonomys swinderianus*). Anim Genet. 2017:48(1):113–115. 10.1111/age.12478. 27436241

[msae071-B2] Altschul SF, Madden TL, Schaffer AA, Zhang J, Zhang Z, Miller W, Lipman DJ. Gapped BLAST and PSI-BLAST: a new generation of protein database search programs. Nucleic Acids Res. 1997:25(17):3389–3402. 10.1093/nar/25.17.3389. 9254694 PMC146917

[msae071-B3] Álvarez A, Arévalo RLM, Verzi DH. Diversification patterns and size evolution in caviomorph rodents. Biol J Linnean Soc. 2017:121(4):907–922. 10.1093/biolinnean/blx026.

[msae071-B4] Antinucci M, Risso D. A matter of taste: lineage-specific loss of function of taste receptor genes in vertebrates. Front Mol Biosci. 2017:4:81. 10.3389/fmolb.2017.00081. 29234667 PMC5712339

[msae071-B5] Barton RA, Harvey PH. Mosaic evolution of brain structure in mammals. Nature 2000:405(6790):1055–1058. 10.1038/35016580. 10890446

[msae071-B6] Barton RA, Purvis A, Harvey PH. Evolutionary radiation of visual and olfactory brain systems in primates, bats and insectivores. Philos Trans R Soc Lond B Biol Sci. 1995:348(1326):381–392. 10.1098/rstb.1995.0076. 7480110

[msae071-B7] Beichman AC, Koepfli KP, Li G, Murphy W, Dobrynin P, Kliver S, Tinker MT, Murray MJ, Johnson J, Lindblad-Toh K, et al Aquatic adaptation and depleted diversity: a deep dive into the genomes of the sea otter and giant otter. Mol Biol Evol. 2019:36(12):2631–2655. 10.1093/molbev/msz101. 31212313 PMC7967881

[msae071-B8] Bell MA, Lloyd GT. strap: an R package for plotting phylogenies against stratigraphy and assessing their stratigraphic congruence. Palaeontology 2015:58(2):379–389. 10.1111/pala.12142.

[msae071-B9] Borowiec ML . AMAS: a fast tool for alignment manipulation and computing of summary statistics. PeerJ 2016:4:e1660. 10.7717/peerj.1660. 26835189 PMC4734057

[msae071-B10] Bouckaert R, Vaughan TG, Barido-Sottani J, Duchene S, Fourment M, Gavryushkina A, Heled J, Jones G, Kuhnert D, De Maio N, et al BEAST 2.5: an advanced software platform for Bayesian evolutionary analysis. PLoS Comput Biol. 2019:15(4):e1006650. 10.1371/journal.pcbi.1006650. 30958812 PMC6472827

[msae071-B11] Brykczynska U, Tzika AC, Rodriguez I, Milinkovitch MC. Contrasted evolution of the vomeronasal receptor repertoires in mammals and squamate reptiles. Genome Biol Evol. 2013:5(2):389–401. 10.1093/gbe/evt013. 23348039 PMC3590772

[msae071-B12] Buck L, Axel R. A novel multigene family may encode odorant receptors: a molecular basis for odor recognition. Cell 1991:65(1):175–187. 10.1016/0092-8674(91)90418-X. 1840504

[msae071-B13] Buffenstein R, Amoroso V, Andziak B, Avdieiev S, Azpurua J, Barker AJ, Bennett NC, Brieno-Enriquez MA, Bronner GN, Coen C, et al The naked truth: a comprehensive clarification and classification of current ‘myths’ in naked mole-rat biology. Biol Rev. 2022:97(1):115–140. 10.1111/brv.12791. 34476892 PMC9277573

[msae071-B14] Burset M, Seledtsov IA, Solovyev VV. Analysis of canonical and non-canonical splice sites in mammalian genomes. Nucleic Acids Res. 2000:28(21):4364–4375. 10.1093/nar/28.21.4364. 11058137 PMC113136

[msae071-B15] Capella-Gutierrez S, Silla-Martinez JM, Gabaldon T. trimAI: a tool for automated alignment trimming in large-scale phylogenetic analyses. Bioinformatics. 2009:25(15):1972–1973. 10.1093/bioinformatics/btp348. 19505945 PMC2712344

[msae071-B16] Carleton MD, Musser GG. Order Rodentia. In: Wilson DE, Reeder DM, editors. Mammal species of the world: a taxonomic and geographic reference. Baltimore, Maryland: Johns Hopkins University Press; 2005. p. 745–752.

[msae071-B17] Chandrashekar J, Hoon MA, Ryba NJ, Zuker CS. The receptors and cells for mammalian taste. Nature 2006:444(7117):288–294. 10.1038/nature05401. 17108952

[msae071-B18] Darriba D, Posada D, Kozlov AM, Stamatakis A, Morel B, Flouri T. ModelTest-NG: a new and scalable tool for the selection of DNA and protein evolutionary models. Mol Biol Evol. 2020:37(1):291–294. 10.1093/molbev/msz189. 31432070 PMC6984357

[msae071-B19] D’Elía G, Fabre P-H, Lessa EP. Rodent systematics in an age of discovery: recent advances and prospects. J Mammal. 2019:100(3):852–871. 10.1093/jmammal/gyy179.

[msae071-B20] Dery TSS, Adenyo C, Kayang BB, Inoue-Murayama M. Assessment of feed resources, management practices and mitigating strategies to feed scarcity in grasscutter (*Thryonomys swinderianus*) production in north-western Ghana. Afr Study Monogr. 2020:40:149–172. 10.14989/250113.

[msae071-B21] Dong D, Jin K, Wu X, Zhong Y. CRDB: database of chemosensory receptor gene families in vertebrate. PLoS One 2012:7(2):e31540. 10.1371/journal.pone.0031540. 22393364 PMC3290609

[msae071-B22] dos Reis M, Inoue J, Hasegawa M, Asher RJ, Donoghue PC, Yang Z. Phylogenomic datasets provide both precision and accuracy in estimating the timescale of placental mammal phylogeny. Proc R Soc B Biol Sci. 2012:279(1742):3491–3500. 10.1098/rspb.2012.0683. PMC3396900 22628470

[msae071-B23] Drummond AJ, Ho SY, Phillips MJ, Rambaut A. Relaxed phylogenetics and dating with confidence. PLoS Biol. 2006:4(5):e88. 10.1371/journal.pbio.0040088. 16683862 PMC1395354

[msae071-B24] Eddy SR . Accelerated profile HMM searches. PLoS Comput Biol. 2011:7(10):e1002195. 10.1371/journal.pcbi.1002195. 22039361 PMC3197634

[msae071-B25] Edler D, Klein J, Antonelli A, Silvestro D. raxmlGUI 2.0: a graphical interface and toolkit for phylogenetic analyses using RAxML. Methods Ecol Evol. 2021:12(2):373–377. 10.1111/2041-210X.13512.

[msae071-B26] Fabre PH, Hautier L, Dimitrov D, Douzery EJ. A glimpse on the pattern of rodent diversification: a phylogenetic approach. BMC Evol Biol. 2012:12(1):88. 10.1186/1471-2148-12-88. 22697210 PMC3532383

[msae071-B27] Felsenstein J . Phylogenies and the comparative method. Am Nat. 1985:125(1):1–15. 10.1086/284325. 31094602

[msae071-B28] Feng P, Zheng J, Rossiter SJ, Wang D, Zhao H. Massive losses of taste receptor genes in toothed and baleen whales. Genome Biol Evol. 2014:6(6):1254–1265. 10.1093/gbe/evu095. 24803572 PMC4079202

[msae071-B29] Francia S, Silvotti L, Ghirardi F, Catzeflis F, Percudani R, Tirindelli R. Evolution of spatially coexpressed families of type-2 vomeronasal receptors in rodents. Genome Biol Evol. 2015:7(1):272–285. 10.1093/gbe/evu283. PMC4316634 25539725

[msae071-B30] Fritz SA, Bininda-Emonds OR, Purvis A. Geographical variation in predictors of mammalian extinction risk: big is bad, but only in the tropics. Ecol Lett. 2009:12(6):538–549. 10.1111/j.1461-0248.2009.01307.x. 19392714

[msae071-B31] Ge D, Wen Z, Xia L, Zhang Z, Erbajeva M, Huang C, Yang Q. Evolutionary history of lagomorphs in response to global environmental change. PLoS One 2013:8(4):e59668. 10.1371/journal.pone.0059668. 23573205 PMC3616043

[msae071-B32] Gilad Y, Przeworski M, Lancet D. Loss of olfactory receptor genes coincides with the acquisition of full trichromatic vision in primates. PLoS Biol. 2004:2(1):E5. 10.1371/journal.pbio.0020005. 14737185 PMC314465

[msae071-B33] Grus WE, Shi P, Zhang YP, Zhang J. Dramatic variation of the vomeronasal pheromone receptor gene repertoire among five orders of placental and marsupial mammals. Proc Natl Acad Sci USA. 2005:102(16):5767–5772. 10.1073/pnas.0501589102. 15790682 PMC556306

[msae071-B34] Grus WE, Zhang J. Distinct evolutionary patterns between chemoreceptors of 2 vertebrate olfactory systems and the differential tuning hypothesis. Mol Biol Evol. 2008:25(8):1593–1601. 10.1093/molbev/msn107. 18460446 PMC2727380

[msae071-B35] Gurevich A, Saveliev V, Vyahhi N, Tesler G. QUAST: quality assessment tool for genome assemblies. Bioinformatics. 2013:29(8):1072–1075. 10.1093/bioinformatics/btt086. 23422339 PMC3624806

[msae071-B36] Gutierrez EA, Castiglione GM, Morrow JM, Schott RK, Loureiro LO, Lim BK, Chang BSW. Functional shifts in bat dim-light visual pigment are associated with differing echolocation abilities and reveal molecular adaptation to photic-limited environments. Mol Biol Evol. 2018:35(10):2422–2434. 10.1093/molbev/msy140. 30010964

[msae071-B37] Hallstrom BM, Janke A. Mammalian evolution may not be strictly bifurcating. Mol Biol Evol. 2010:27(12):2804–2816. 10.1093/molbev/msq166. 20591845 PMC2981514

[msae071-B38] Hara Y, Tatsumi K, Yoshida M, Kajikawa E, Kiyonari H, Kuraku S. Optimizing and benchmarking de novo transcriptome sequencing: from library preparation to assembly evaluation. BMC Genomics. 2015:16(1):977. 10.1186/s12864-015-2007-1. 26581708 PMC4652379

[msae071-B39] Hayakawa T, Suzuki-Hashido N, Matsui A, Go Y. Frequent expansions of the bitter taste receptor gene repertoire during evolution of mammals in the Euarchontoglires clade. Mol Biol Evol. 2014:31(8):2018–2031. 10.1093/molbev/msu144. 24758778

[msae071-B40] Hayden S, Bekaert M, Crider TA, Mariani S, Murphy WJ, Teeling EC. Ecological adaptation determines functional mammalian olfactory subgenomes. Genome Res. 2010:20(1):1–9. 10.1101/gr.099416.109. 19952139 PMC2798820

[msae071-B41] Hayden S, Bekaert M, Goodbla A, Murphy WJ, Dávalos LM, Teeling EC. A cluster of olfactory receptor genes linked to frugivory in bats. Mol Biol Evol. 2014:31(4):917–927. 10.1093/molbev/msu043. 24441035

[msae071-B42] Hohenbrink P, Mundy NI, Zimmermann E, Radespiel U. First evidence for functional vomeronasal 2 receptor genes in primates. Biol Lett. 2013:9(1):20121006. 10.1098/rsbl.2012.1006. 23269843 PMC3565523

[msae071-B43] Honeycutt R . Rodents (Rodentia). In: Hedges SB, Kumar S, editors. The timetree of life. Oxford.: Oxford University Press; 2009. p. 492–494.

[msae071-B44] Jameson NM, Hou ZC, Sterner KN, Weckle A, Goodman M, Steiper ME, Wildman DE. Genomic data reject the hypothesis of a prosimian primate clade. J Hum Evol. 2011:61(3):295–305. 10.1016/j.jhevol.2011.04.004. 21620437

[msae071-B45] Jarvis JUM . Eusociality in a mammal: cooperative breeding in naked mole-rat colonies. Science 1981:212(4494):571–573. 10.1126/science.7209555. 7209555

[msae071-B46] Jarvis JUM, Bennett NC. Eusociality has evolved independently in two genera of bathyergid mole-rats—but occurs in no other subterranean mammal. Behav Ecol Sociobiol. 1993:33(4):253–260. 10.1007/BF02027122.

[msae071-B47] Jiang P, Josue J, Li X, Glaser D, Li W, Brand JG, Margolskee RF, Reed DR, Beauchamp GK. Major taste loss in carnivorous mammals. Proc Natl Acad Sci USA. 2012:109(13):4956–4961. 10.1073/pnas.1118360109. 22411809 PMC3324019

[msae071-B48] Kalyaanamoorthy S, Minh BQ, Wong TKF, von Haeseler A, Jermiin LS. ModelFinder: fast model selection for accurate phylogenetic estimates. Nat Methods. 2017:14(6):587–589. 10.1038/nmeth.4285. 28481363 PMC5453245

[msae071-B49] Katoh K, Standley DM. MAFFT multiple sequence alignment software version 7: improvements in performance and usability. Mol Biol Evol. 2013:30(4):772–780. 10.1093/molbev/mst010. 23329690 PMC3603318

[msae071-B50] Keesey IW, Grabe V, Gruber L, Koerte S, Obiero GF, Bolton G, Khallaf MA, Kunert G, Lavista-Llanos S, Valenzano DR, et al Inverse resource allocation between vision and olfaction across the genus Drosophila. Nat Commun. 2019:10(1):1162. 10.1038/s41467-019-09087-z. 30858374 PMC6411718

[msae071-B51] Kessler DS, Hope K, Maslanka M. Behavior, nutrition, and veterinary care of patagonian cavies (*Dolichotis patagonum*). Vet Clin North Am Exot Anim Pract. 2009:12(2):267–278. 10.1016/j.cvex.2009.01.009. 19341953

[msae071-B52] Kishida T . Olfaction of aquatic amniotes. Cell Tissue Res. 2021:383(1):353–365. 10.1007/s00441-020-03382-8. 33409651

[msae071-B53] Kishida T, Thewissen JG. Evolutionary changes of the importance of olfaction in cetaceans based on the olfactory marker protein gene. Gene 2012:492(2):349–353. 10.1016/j.gene.2011.11.013. 22123181

[msae071-B54] Kishida T, Thewissen JG, Hayakawa T, Imai H, Agata K. Aquatic adaptation and the evolution of smell and taste in whales. Zool Lett. 2015:1(1):9. 10.1186/s40851-014-0002-z. PMC4604112 26605054

[msae071-B55] Kriventseva EV, Kuznetsov D, Tegenfeldt F, Manni M, Dias R, Simão FA, Zdobnov EM. OrthoDB v10: sampling the diversity of animal, plant, fungal, protist, bacterial and viral genomes for evolutionary and functional annotations of orthologs. Nucleic Acids Res. 2019:47(D1):D807–D811. 10.1093/nar/gky1053. 30395283 PMC6323947

[msae071-B56] Krogh A, Larsson B, von Heijne G, Sonnhammer EL. Predicting transmembrane protein topology with a hidden Markov model: application to complete genomes. J Mol Biol. 2001:305(3):567–580. 10.1006/jmbi.2000.4315. 11152613

[msae071-B57] Kumar S, Stecher G, Suleski M, Hedges SB. TimeTree: a resource for timelines, timetrees, and divergence times. Mol Biol Evol. 2017:34(7):1812–1819. 10.1093/molbev/msx116. 28387841

[msae071-B58] Lacher TE, Murphy WJ, Rogan J, Smith AT, Upham NS, Wilson DE Evolution, phylogeny, ecology, and conservation of the clade glires: lagomorpha and rodentia. In: Wilson DE, editor. Handbook of the mammals of the world: volume 6 lagomorphs and rodents I. Barcelona: Lynx Edicions; 2016. p. 15–26.

[msae071-B59] Lanfear R, Calcott B, Kainer D, Mayer C, Stamatakis A. Selecting optimal partitioning schemes for phylogenomic datasets. BMC Evol Biol. 2014:14(1):82. 10.1186/1471-2148-14-82. 24742000 PMC4012149

[msae071-B60] Lanfear R, Frandsen PB, Wright AM, Senfeld T, Calcott B. PartitionFinder 2: new methods for selecting partitioned models of evolution for molecular and morphological phylogenetic analyses. Mol Biol Evol. 2017:34(3):772–773. 10.1093/molbev/msw260. 28013191

[msae071-B61] Laurin M, Lapauze O, Marjanović D. What do ossification sequences tell us about the origin of extant amphibians? Peer Community J. 2022:2:e12. 10.24072/pcjournal.89.

[msae071-B62] Levy Karin E, Mirdita M, Söding J. MetaEuk-sensitive, high-throughput gene discovery, and annotation for large-scale eukaryotic metagenomics. Microbiome 2020:8(1):48. 10.1186/s40168-020-00808-x. 32245390 PMC7126354

[msae071-B63] Li D, Zhang J. Diet shapes the evolution of the vertebrate bitter taste receptor gene repertoire. Mol Biol Evol. 2014:31(2):303–309. 10.1093/molbev/mst219. 24202612 PMC3907052

[msae071-B64] Liberles SD . Mammalian pheromones. Annu Rev Physiol. 2014:76(1):151–175. 10.1146/annurev-physiol-021113-170334. 23988175 PMC4310675

[msae071-B65] Liu A, He F, Shen L, Liu R, Wang Z, Zhou J. Convergent degeneration of olfactory receptor gene repertoires in marine mammals. BMC Genomics. 2019:20(1):977. 10.1186/s12864-019-6290-0. 31842731 PMC6916060

[msae071-B66] Manni M, Berkeley MR, Seppey M, Simão FA, Zdobnov EM. BUSCO update: novel and streamlined workflows along with broader and deeper phylogenetic coverage for scoring of eukaryotic, prokaryotic, and viral genomes. Mol Biol Evol. 2021:38(10):4647–4654. 10.1093/molbev/msab199. 34320186 PMC8476166

[msae071-B67] Matsui A, Go Y, Niimura Y. Degeneration of olfactory receptor gene repertories in primates: no direct link to full trichromatic vision. Mol Biol Evol. 2010:27(5):1192–1200. 10.1093/molbev/msq003. 20061342

[msae071-B68] Minh BQ, Schmidt HA, Chernomor O, Schrempf D, Woodhams MD, von Haeseler A, Lanfear R. IQ-TREE 2: new models and efficient methods for phylogenetic inference in the genomic era. Mol Biol Evol. 2020:37(5):1530–1534. 10.1093/molbev/msaa015. 32011700 PMC7182206

[msae071-B69] Nei M, Niimura Y, Nozawa M. The evolution of animal chemosensory receptor gene repertoires: roles of chance and necessity. Nat Rev Genet. 2008:9(12):951–963. 10.1038/nrg2480. 19002141

[msae071-B70] Niimura Y . Olfactory receptor multigene family in vertebrates: from the viewpoint of evolutionary genomics. Curr Genomics. 2012:13(2):103–114. 10.2174/138920212799860706. 23024602 PMC3308321

[msae071-B71] Niimura Y . Identification of olfactory receptor genes from mammalian genome sequences. Methods Mol Biol. 2013:1003:39–49. 10.1007/978-1-62703-377-0_3. 23585032

[msae071-B72] Niimura Y, Ihara S, Touhara K. Mammalian olfactory and vomeronasal recetor families. In: Fritzsch B, editor. The senses: A comprehensive reference. 2nd ed. Amsterdam: Elsevier; 2020. p. 516–535.

[msae071-B73] Niimura Y, Matsui A, Touhara K. Extreme expansion of the olfactory receptor gene repertoire in African elephants and evolutionary dynamics of orthologous gene groups in 13 placental mammals. Genome Res. 2014:24(9):1485–1496. 10.1101/gr.169532.113. 25053675 PMC4158756

[msae071-B74] Niimura Y, Matsui A, Touhara K. Acceleration of olfactory receptor gene loss in primate evolution: possible link to anatomical change in sensory systems and dietary transition. Mol Biol Evol. 2018:35(6):1437–1450. 10.1093/molbev/msy042. 29659972

[msae071-B75] Niimura Y, Nei M. Evolution of olfactory receptor genes in the human genome. Proc Natl Acad Sci USA. 2003:100(21):12235–12240. 10.1073/pnas.1635157100. 14507991 PMC218742

[msae071-B76] Niimura Y, Nei M. Extensive gains and losses of olfactory receptor genes in mammalian evolution. PLoS One 2007:2(8):e708. 10.1371/journal.pone.0000708. 17684554 PMC1933591

[msae071-B77] Niimura Y, Tsunoda M, Kato S, Murata K, Yanagawa T, Suzuki S, Touhara K. Origin and evolution of the gene family of proteinaceous pheromones, the exocrine gland-secreting peptides, in rodents. Mol Biol Evol. 2021:38(2):634–649. 10.1093/molbev/msaa220. 32961551 PMC7826187

[msae071-B78] Niven JE, Laughlin SB. Energy limitation as a selective pressure on the evolution of sensory systems. J Exp Biol. 2008:211(11):1792–1804. 10.1242/jeb.017574. 18490395

[msae071-B79] Oosthuizen MK, Cooper HM, Bennett NC. Circadian rhythms of locomotor activity in solitary and social species of African mole-rats (family: Bathyergidae). J Biol Rhythms. 2003:18(6):481–490. 10.1177/0748730403259109. 14667149

[msae071-B80] Parra G, Bradnam K, Korf I. CEGMA: a pipeline to accurately annotate core genes in eukaryotic genomes. Bioinformatics. 2007:23(9):1061–1067. 10.1093/bioinformatics/btm071. 17332020

[msae071-B81] Pozzi L, Hodgson JA, Burrell AS, Sterner KN, Raaum RL, Disotell TR. Primate phylogenetic relationships and divergence dates inferred from complete mitochondrial genomes. Mol Phylogenet Evol. 2014:75:165–183. 10.1016/j.ympev.2014.02.023. 24583291 PMC4059600

[msae071-B82] Rambaut A, Drummond AJ, Xie D, Baele G, Suchard MA. Posterior summarization in Bayesian phylogenetics using Tracer 1.7. Syst Biol. 2018:67(5):901–904. 10.1093/sysbio/syy032. 29718447 PMC6101584

[msae071-B83] Rodriguez I, Greer CA, Mok MY, Mombaerts P. A putative pheromone receptor gene expressed in human olfactory mucosa. Nat Genet. 2000:26(1):18–19. 10.1038/79124. 10973240

[msae071-B84] Saitou N, Nei M. The neighbor-joining method: a new method for reconstructing phylogenetic trees. Mol Biol Evol. 1987:4(4):406–425. 10.1093/oxfordjournals.molbev.a040454. 3447015

[msae071-B85] Shang S, Wu X, Chen J, Zhang H, Zhong H, Wei Q, Yan J, Li H, Liu G, Sha W, et al The repertoire of bitter taste receptor genes in canids. Amino Acids. 2017:49(7):1159–1167. 10.1007/s00726-017-2422-5. 28417226

[msae071-B86] Shao Y, Li JX, Ge RL, Zhong L, Irwin DM, Murphy RW, Zhang YP. Genetic adaptations of the plateau zokor in high-elevation burrows. Sci Rep. 2015:5(1):17262. 10.1038/srep17262. 26602147 PMC4658562

[msae071-B87] Shen B, Fang T, Dai M, Jones G, Zhang S. Independent losses of visual perception genes Gja10 and Rbp3 in echolocating bats (order: Chiroptera). PLoS One 2013:8(7):e68867. 10.1371/journal.pone.0068867. 23874796 PMC3715546

[msae071-B88] Shi P, Zhang J. Contrasting modes of evolution between vertebrate sweet/umami receptor genes and bitter receptor genes. Mol Biol Evol. 2006:23(2):292–300. 10.1093/molbev/msj028. 16207936

[msae071-B89] Sievers F, Wilm A, Dineen D, Gibson TJ, Karplus K, Li W, Lopez R, McWilliam H, Remmert M, Söding J, et al Fast, scalable generation of high-quality protein multiple sequence alignments using Clustal Omega. Mol Syst Biol. 2011:7(1):539. 10.1038/msb.2011.75. 21988835 PMC3261699

[msae071-B90] Silvotti L, Cavalca E, Gatti R, Percudani R, Tirindelli R. A recent class of chemosensory neurons developed in mouse and rat. PLoS One 2011:6(9):e24462. 10.1371/journal.pone.0024462. 21931725 PMC3170373

[msae071-B91] Smythe N . The natural history of the Central American agouti (*Dasyprocta punctata*). Smithsonian Contrib Zool. 1978:157(257):1–52. 10.5479/si.00810282.257.

[msae071-B92] Springer MS, Gatesy J. Inactivation of the olfactory marker protein (OMP) gene in river dolphins and other odontocete cetaceans. Mol Phylogenet Evol. 2017:109:375–387. 10.1016/j.ympev.2017.01.020. 28193458

[msae071-B93] Stamatakis A . RAxML version 8: a tool for phylogenetic analysis and post-analysis of large phylogenies. Bioinformatics. 2014:30(9):1312–1313. 10.1093/bioinformatics/btu033. 24451623 PMC3998144

[msae071-B94] Stöckl A, Heinze S, Charalabidis A, El Jundi B, Warrant E, Kelber A. Differential investment in visual and olfactory brain areas reflects behavioural choices in hawk moths. Sci Rep. 2016:6(1):26041. 10.1038/srep26041. 27185464 PMC4869021

[msae071-B95] Takami S . Recent progress in the neurobiology of the vomeronasal organ. Microsc Res Tech. 2002:58(3):228–250. 10.1002/jemt.10094. 12203701

[msae071-B96] Takezaki N, Rzhetsky A, Nei M. Phylogenetic test of the molecular clock and linearized trees. Mol Biol Evol. 1995:12(5):823–833. 10.1093/oxfordjournals.molbev.a040259. 7476128

[msae071-B97] Tirindelli R . Coding of pheromones by vomeronasal receptors. Cell Tissue Res. 2021:383(1):367–386. 10.1007/s00441-020-03376-6. 33433690

[msae071-B98] Upham NS, Patterson BD. Diversification and biogeography of the Neotropical caviomorph lineage Octodontoidea (Rodentia: Hystricognathi). Mol Phylogenet Evol. 2012:63(2):417–429. 10.1016/j.ympev.2012.01.020. 22327013

[msae071-B99] Upham NS, Patterson BD. Phylogeny and evolution of caviomorph rodents: a complete phylogeny and timetree for living genera. In: VAA D, editors. Biology of caviomorph rodents: diversity and evolution. Buenos Aires, Argentina: Sociedad Argentina para el Estudio de los Mamíferos (SAREM); 2015. p. 63–120.

[msae071-B100] Vurture GW, Sedlazeck FJ, Nattestad M, Underwood CJ, Fang H, Gurtowski J, Schatz MC. GenomeScope: fast reference-free genome profiling from short reads. Bioinformatics. 2017:33(14):2202–2204. 10.1093/bioinformatics/btx153. 28369201 PMC5870704

[msae071-B101] Wilman H, Belmaker J, Simpson J, de la Rosa C, Rivadeneira MM, Jetz W. EltonTraits 1.0: species-level foraging attributes of the world’s birds and mammals. Ecology. 2014:95(7):2027. 10.1890/13-1917.1.

[msae071-B102] Wolsan M, Sato JJ. Role of feeding specialization in taste receptor loss: insights from sweet and umami receptor evolution in Carnivora. Chem Senses. 2022:47:bjac033. 10.1093/chemse/bjac033. 36433799 PMC9680018

[msae071-B103] Wu J, Jiao H, Simmons NB, Lu Q, Zhao H. Testing the sensory trade-off hypothesis in New World bats. Proc Biol Sci. 2018:285(1885):20181523. 10.1098/rspb.2018.1523. 30158315 PMC6125922

[msae071-B104] Young JM, Massa HF, Hsu L, Trask BJ. Extreme variability among mammalian V1R gene families. Genome Res. 2010:20(1):10–18. 10.1101/gr.098913.109. 19952141 PMC2798821

[msae071-B105] Young JM, Trask BJ. V2r gene families degenerated in primates, dog and cow, but expanded in opossum. Trends Genet. 2007:23(5):212–215. 10.1016/j.tig.2007.03.004. 17382427

[msae071-B106] Zhang Z, Nikaido M. Inactivation of ancV1R as a predictive signature for the loss of vomeronasal system in mammals. Genome Biol Evol. 2020:12(6):766–778. 10.1093/gbe/evaa082. 32315408 PMC7290294

[msae071-B107] Zhao H, Rossiter SJ, Teeling EC, Li C, Cotton JA, Zhang S. The evolution of color vision in nocturnal mammals. Proc Natl Acad Sci USA. 2009:106(22):8980–8985. 10.1073/pnas.0813201106. 19470491 PMC2690009

[msae071-B108] Zhao H, Xu D, Zhang S, Zhang J. Genomic and genetic evidence for the loss of umami taste in bats. Genome Biol Evol. 2012:4(1):73–79. 10.1093/gbe/evr126. 22117084 PMC3318850

[msae071-B109] Zhu K, Zhou X, Xu S, Sun D, Ren W, Zhou K, Yang G. The loss of taste genes in cetaceans. BMC Evol Biol. 2014:14(1):218. doi:10.1186/s12862-014-0218-8. 25305673 PMC4232718

