# Supplementary material for: Synchronized Expansion and Contraction of Olfactory, Vomeronasal, and Taste Receptor Gene Families in Hystricomorph Rodents
Source: Mol Biol Evol. 2024 Apr 23;41(4):msae071. doi: 10.1093/molbev/msae071 (PMC11035023; doi:10.1093/molbev/msae071)
Supplement: msae071_Supplementary_Data [file msae071_supplementary_data.zip › Supplementary_Figures.pdf]

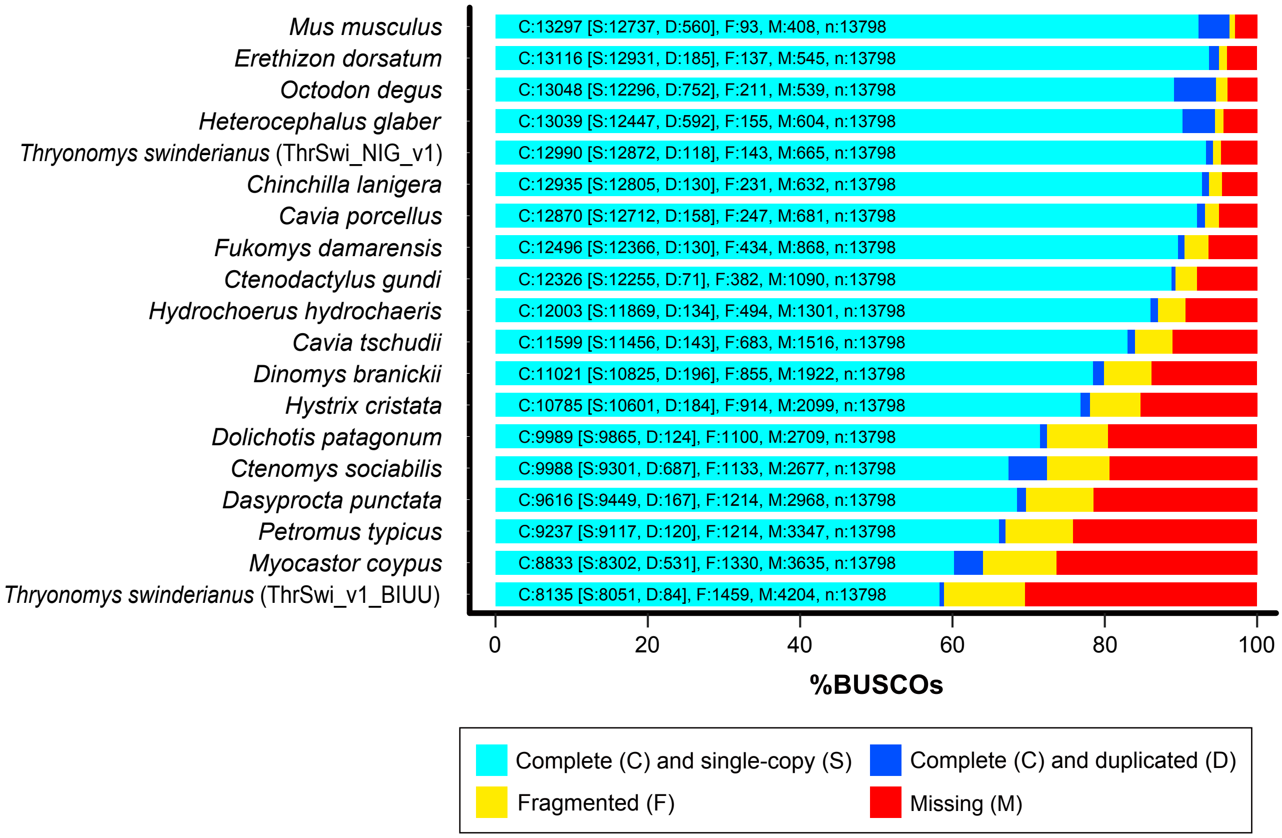

Fig. S1. Comparison of BUSCO results among genome assemblies used in this study.

The numbers in each bar indicate as follows. C, complete genes; S, single-copy genes; D, duplicated genes; F, fragmented genes; M, missing genes; n, total number of genes in BUSCO glires database used in this analysis.

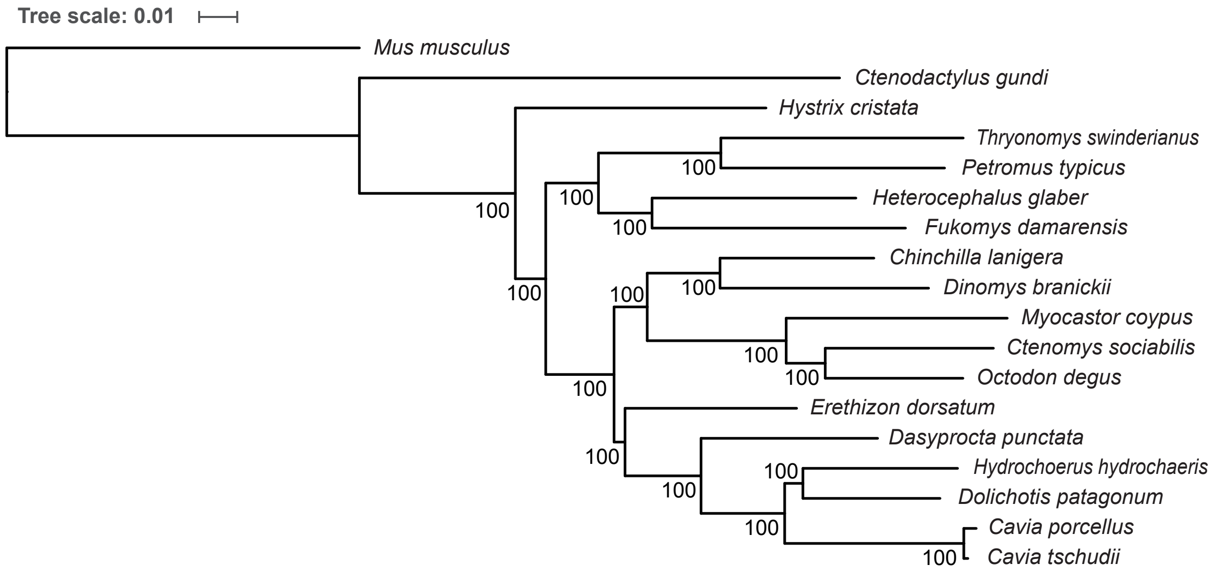

Fig. S2. Maximum likelihood tree indicating the phylogenetic position of each species used in this study.

The scale bar represents 0.01 amino acid substitutions per site. The number at each node indicates the maximum likelihood bootstrap value in percentage.

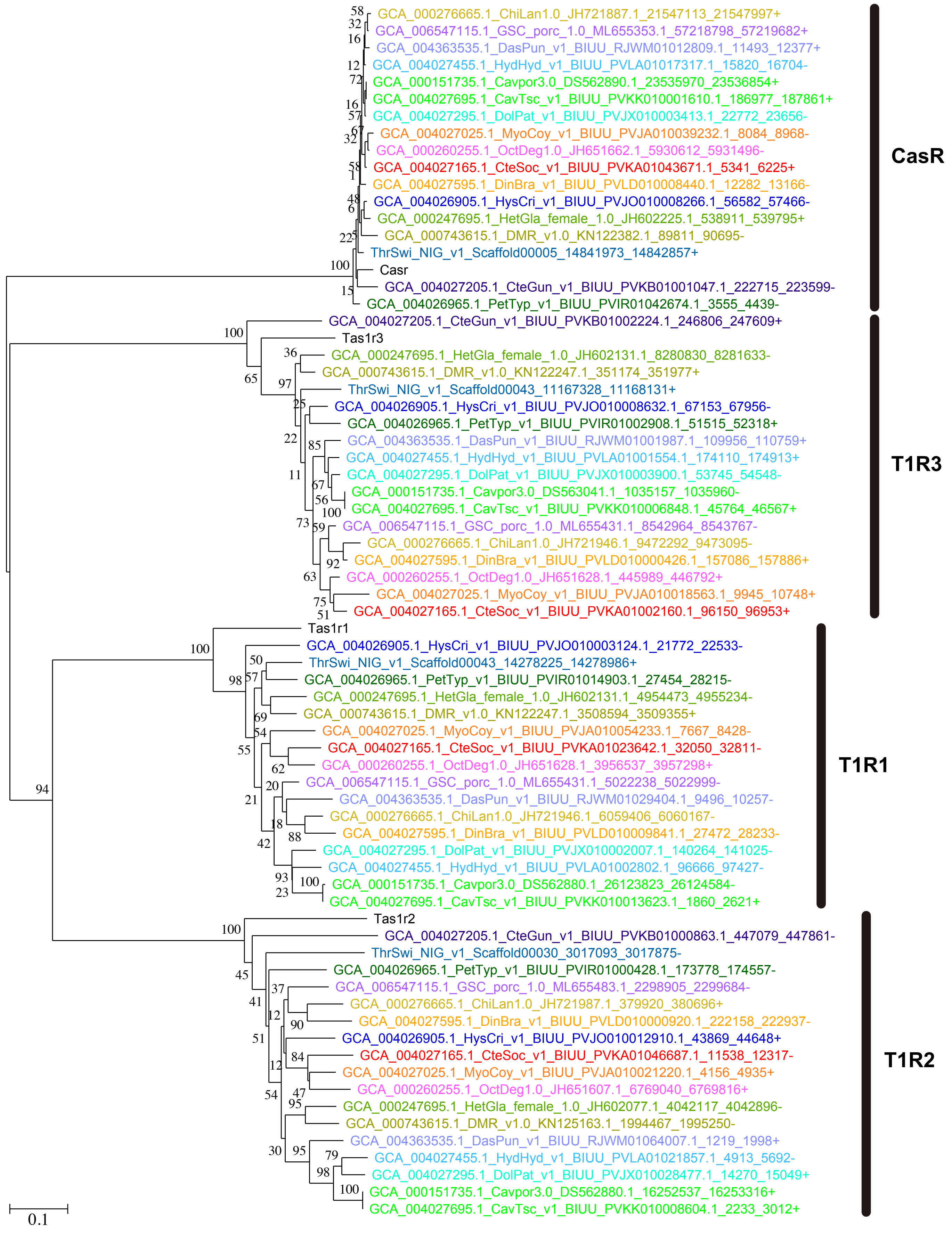

Fig. S3. NJ phylogenetic tree for exon 3 seqeunces of T1R genes from 17 Hystricomorpha species and mice with CasR sequences as outgroups. A bootstrap value obtained from 500 resamplings is shown at each node. Each gene is colored according to the color code in Figs. 3 and 4.

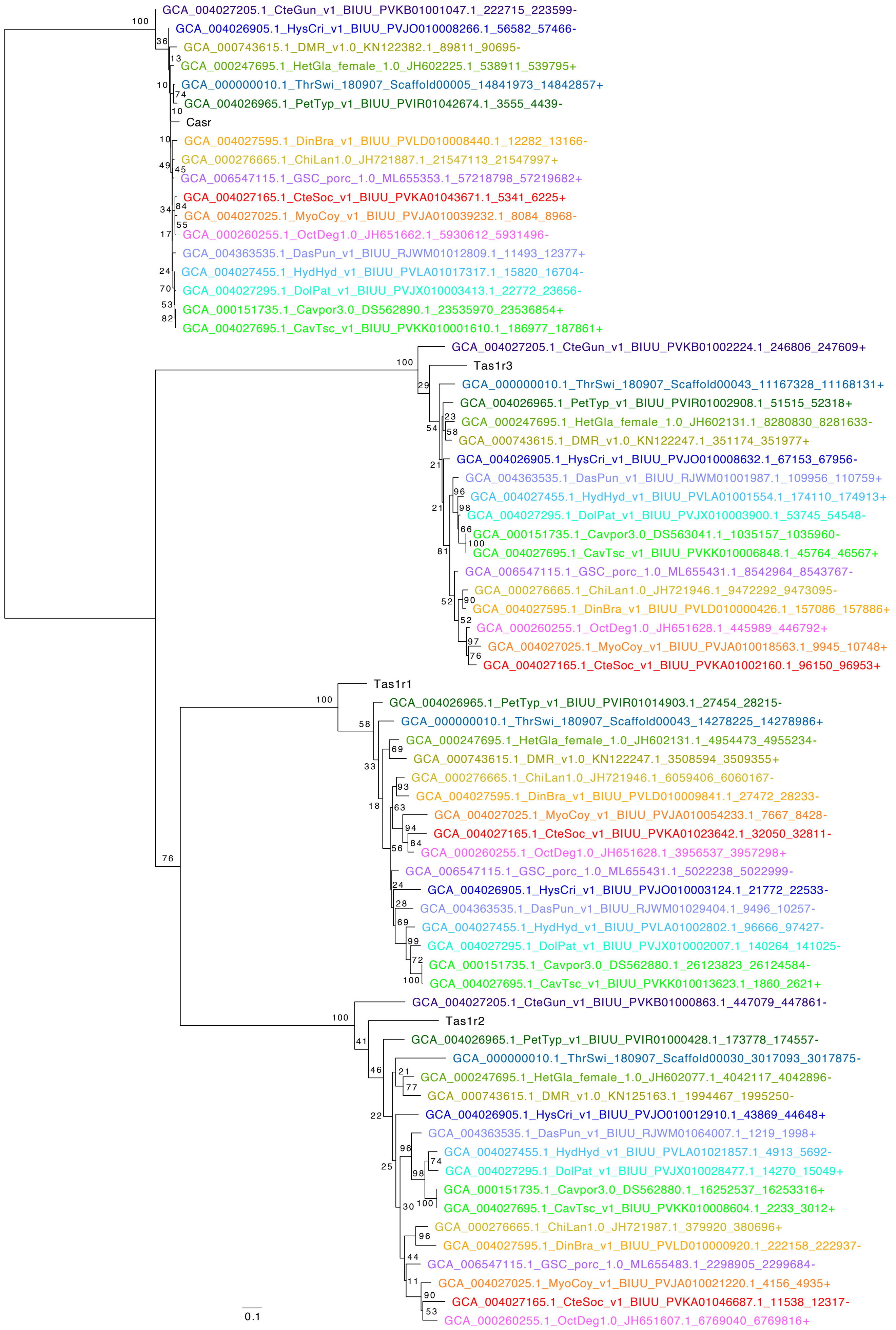

Fig. S4. ML phylogenetic tree for T1R genes.

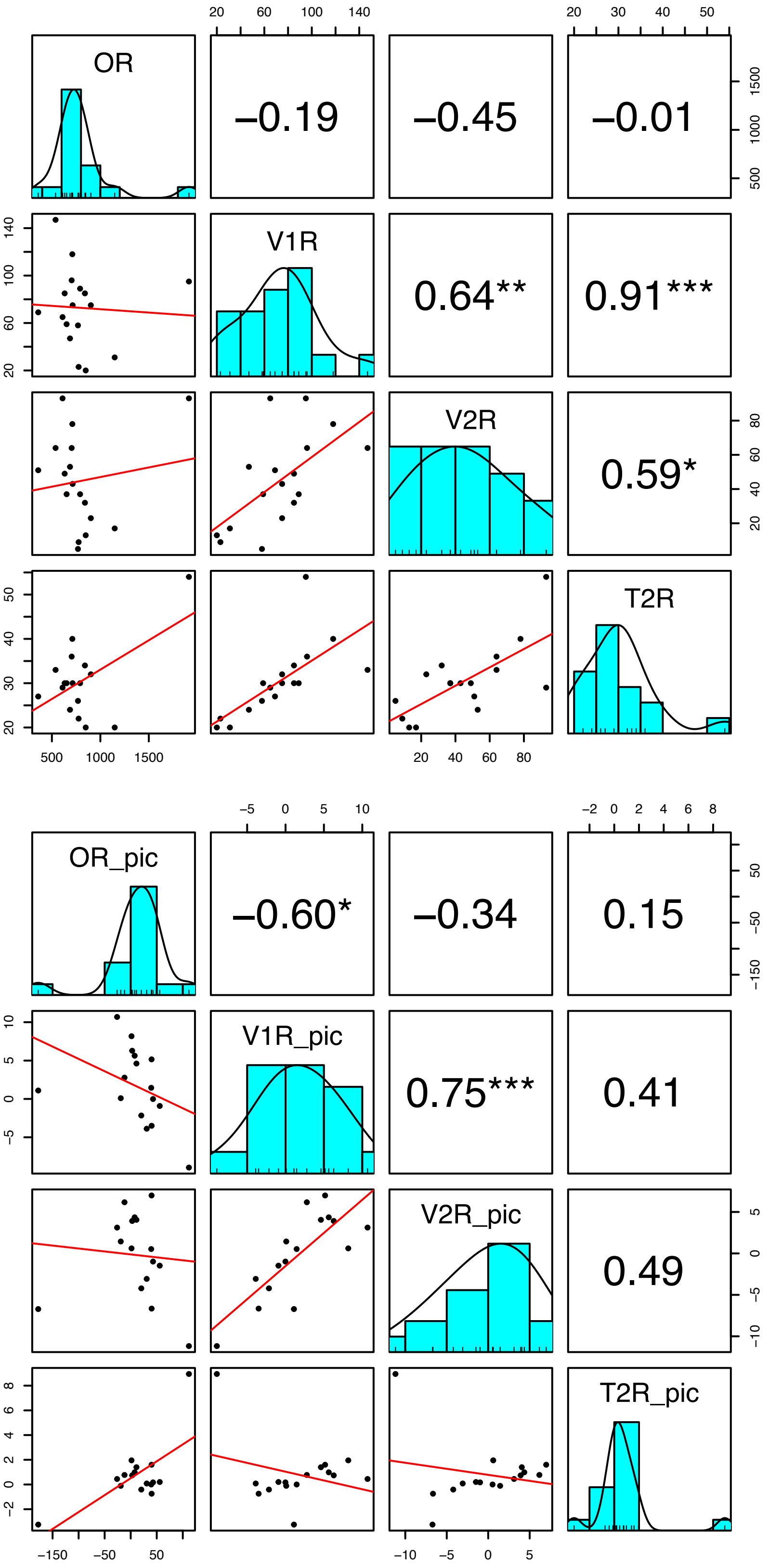

Fig. S5. Correlation of the numbers of intact genes among OR, V1R, V2R, and T2R genes.

Top and bottom diagrams show the results before and after removing phylogenetic dependence, respectively. “pic” indicates phylogenetically independent constant. A number in each square indicates the Spearman correlation coefficient. *, $p < 5\%$; **, $p < 1\%$; ***, $p < 0.1\%$.

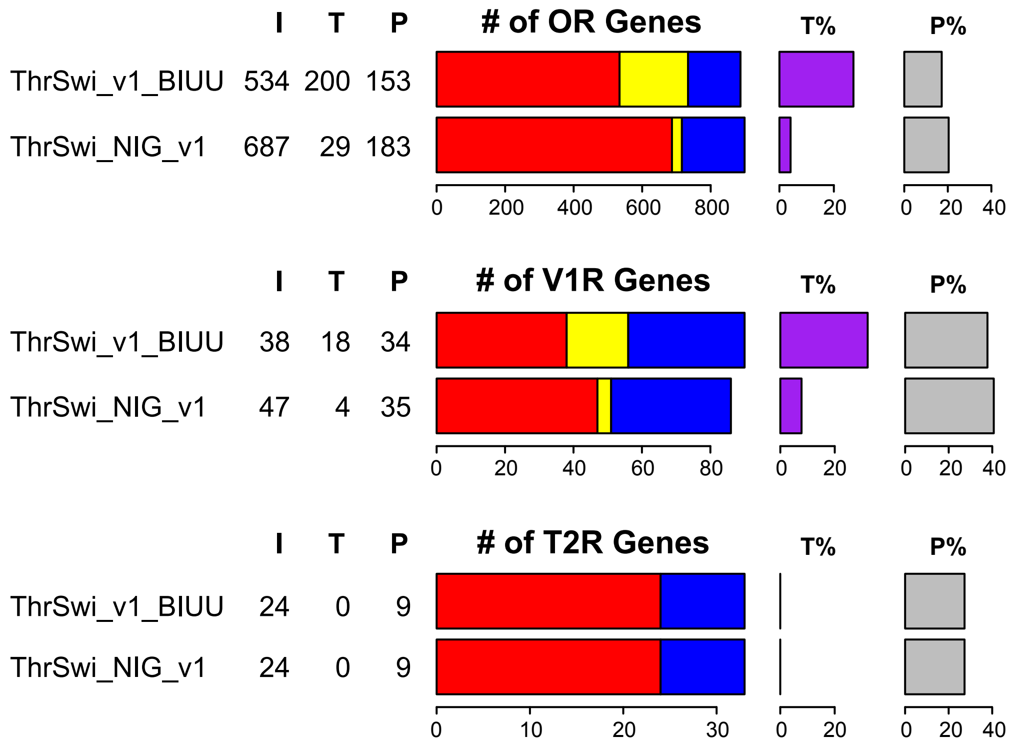

Fig. S6. Comparison of the numbers of genes identified from two genome assemblies of the grasscutter, ThrSwi_v1_BIUU and ThrSwi_NIG_v1 (this study) for OR, V1R, and T2R genes.

Red, yellow, and blue bars indicate the numbers of intact genes (I), truncated genes (T) and pseudogenes (P), respectively.

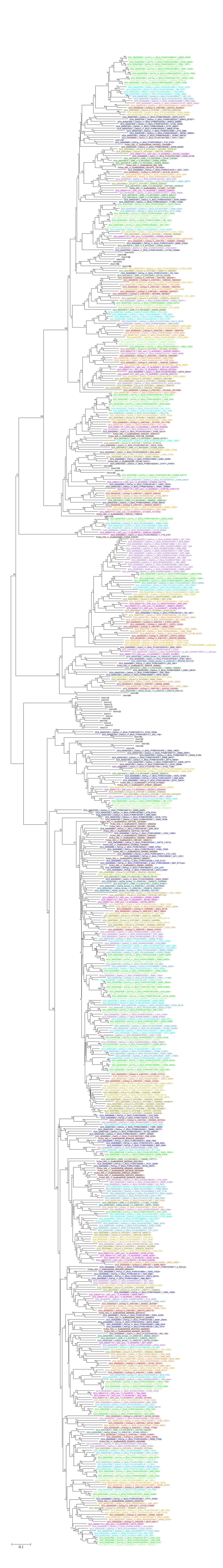

Fig. S7. NJ phylogenetic tree for V1R-1 genes.

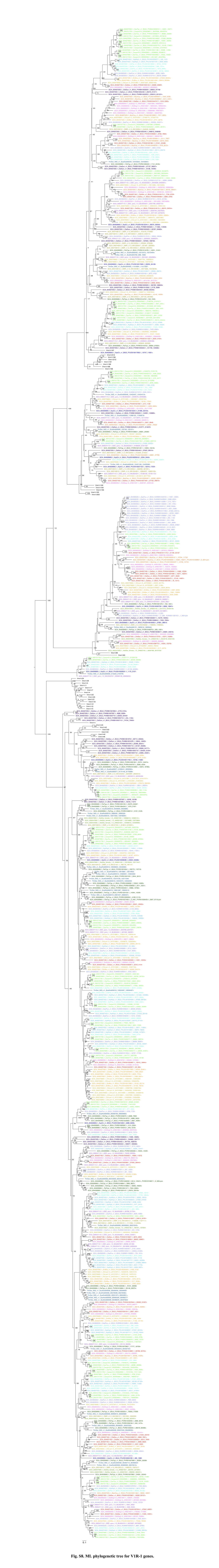

Fig. S8. ML phylogenetic tree for V1R-1 genes.

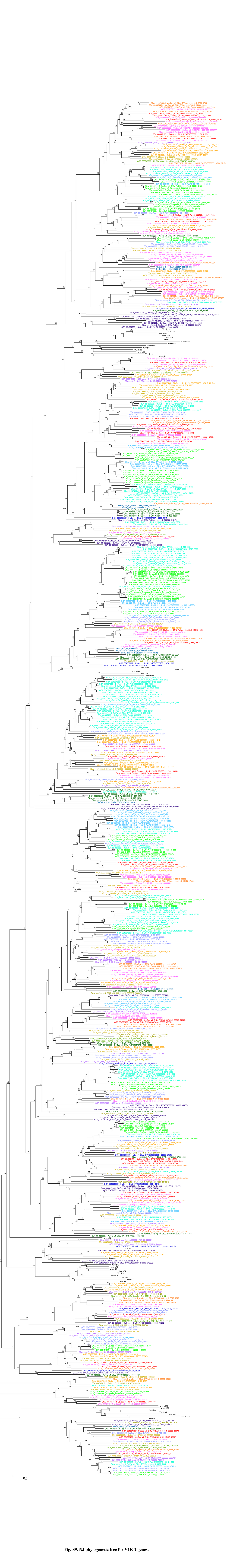

Fig. S9. NJ phylogenetic tree for V1R-2 genes.

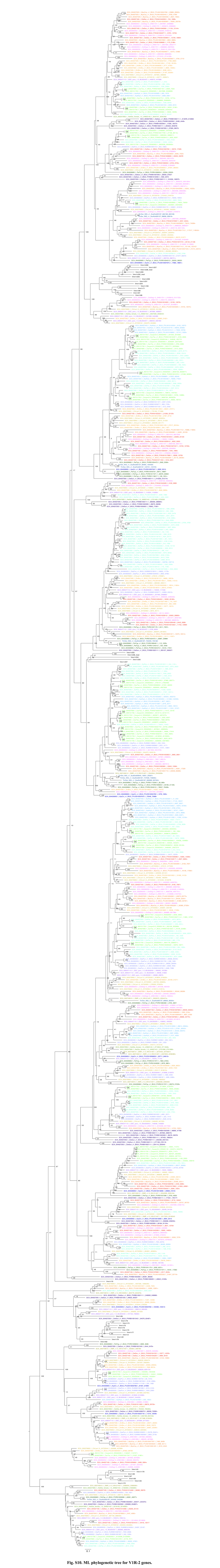

Fig. S10. ML phylogenetic tree for V1R-2 genes.

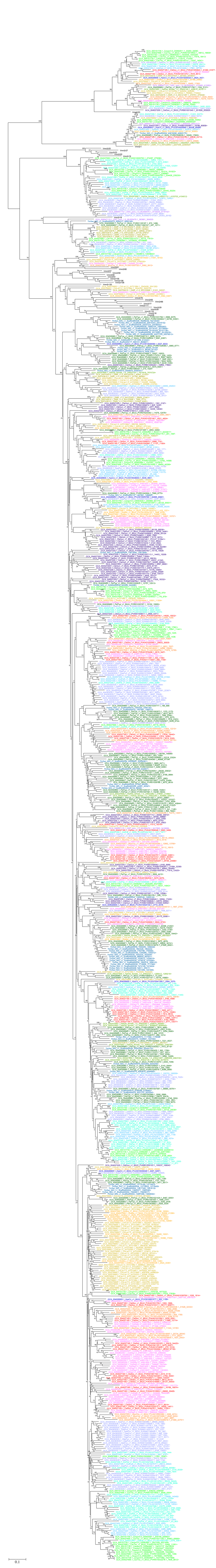

Fig. S11. NJ phylogenetic tree for exon 3 sequences of V2R genes.

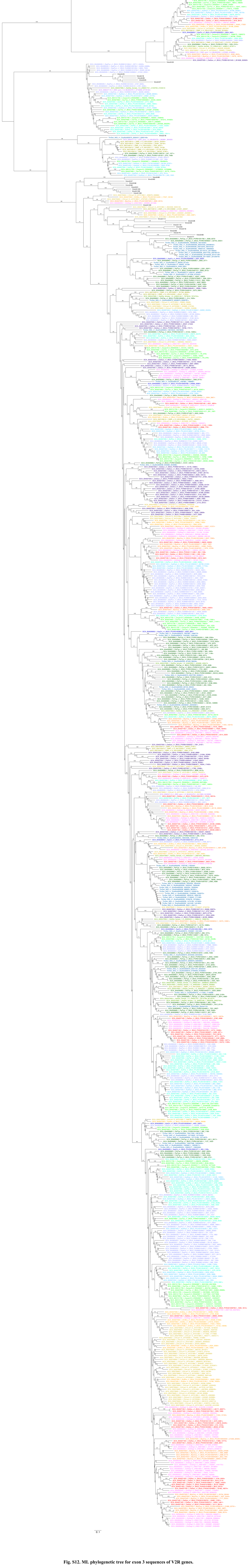

Fig. S12. ML phylogenetic tree for exon 3 sequences of V2R genes.

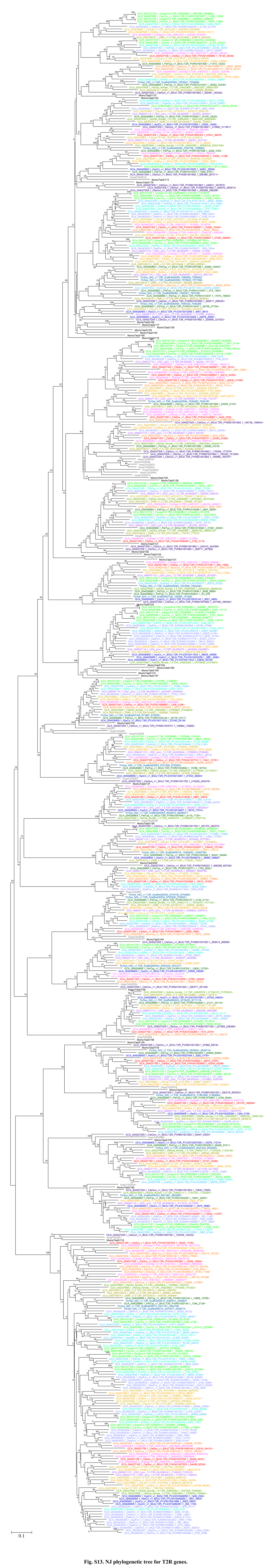

Fig. S13. NJ phylogenetic tree for T2R genes.

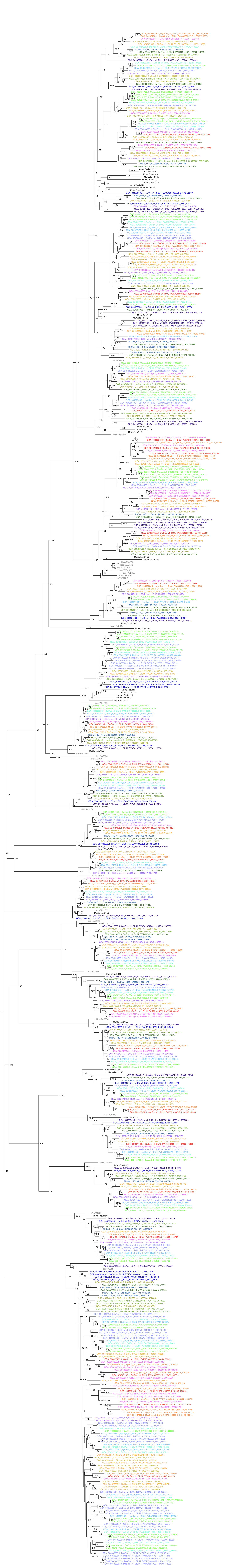

Fig. S14. ML phylogenetic tree for T2R genes.

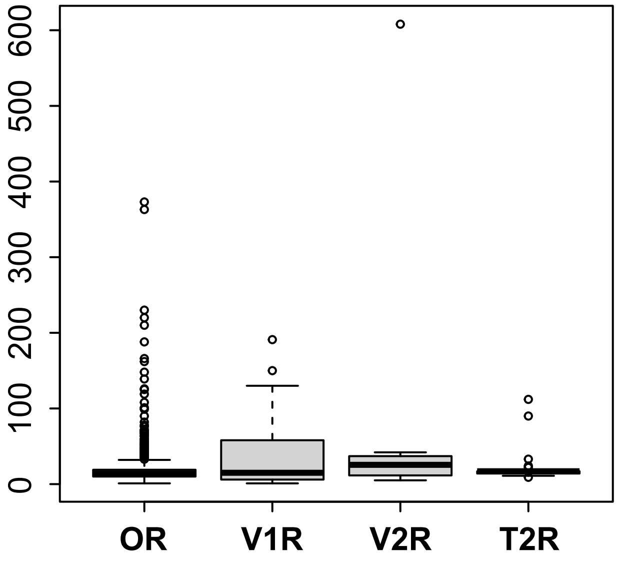

Fig. S15. Comparison of the numbers of genes belonging to each OGG among four gene families.

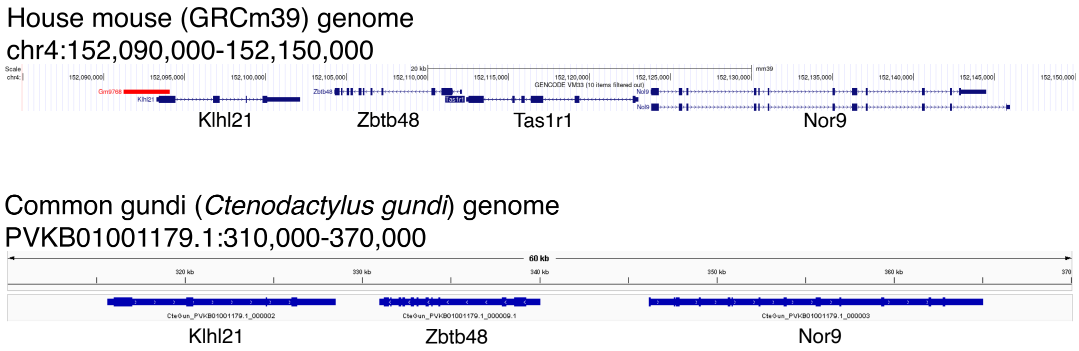

Fig. S16. Genomic regions encoding the T1R1 gene and its flanking genes of the mouse (top) and the common gundi (bottom).

The genomic region of the mouse GRCm39, chr4: 152,090,000-152,150,000, was obtained from the USCS Genome Browser. The genomic region of the common gundi, PVKB01001179.1: 310,000-370,000 was visualized using the Integrative Genomics Viewer (IGV). The gene positions of the predicted sequences of *Klhl21*, *Zbtb48*, and *Nor9* in the common gundi are also indicated.

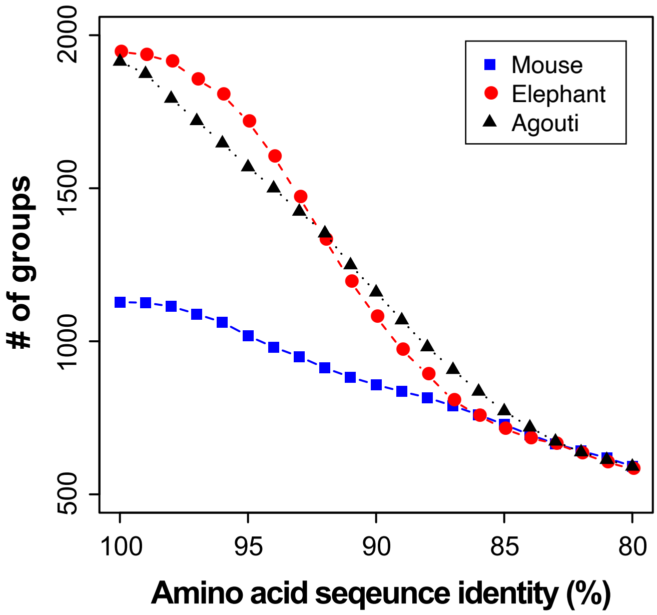

Fig. S17. OR genes in the Central American agouti genome is as diverse as those in the African elephant genome.

We first calculated all-against-all amino acid sequence identities after eliminating gaps between any pairs of OR genes in each species. Then, all OR genes in each species are classified into groups with a given amino acid sequence identity threshold, *A* (%). If a gene shows an identity >*A*% to at least one member gene belonging to a group, then the gene is added to the group. For example, let us consider three OR genes, OR1, OR2, and OR3. Suppose that the amino acid seqeunce identities of OR1-OR2, OR1-OR3, and OR2-OR3 are 91%, 92%, and 88%, respectively. The three genes are classified into one group with a 90% threshold (though the identity between OR2 and OR3 is less than the threshold). 1,948, 1,914, and 1,130 OR genes in the Central American agouti, African elephant, and mouse, respectively, were examined. Amino acid sequences of the African elephant and mouse OR genes were taken from Niimura et al. (2014).

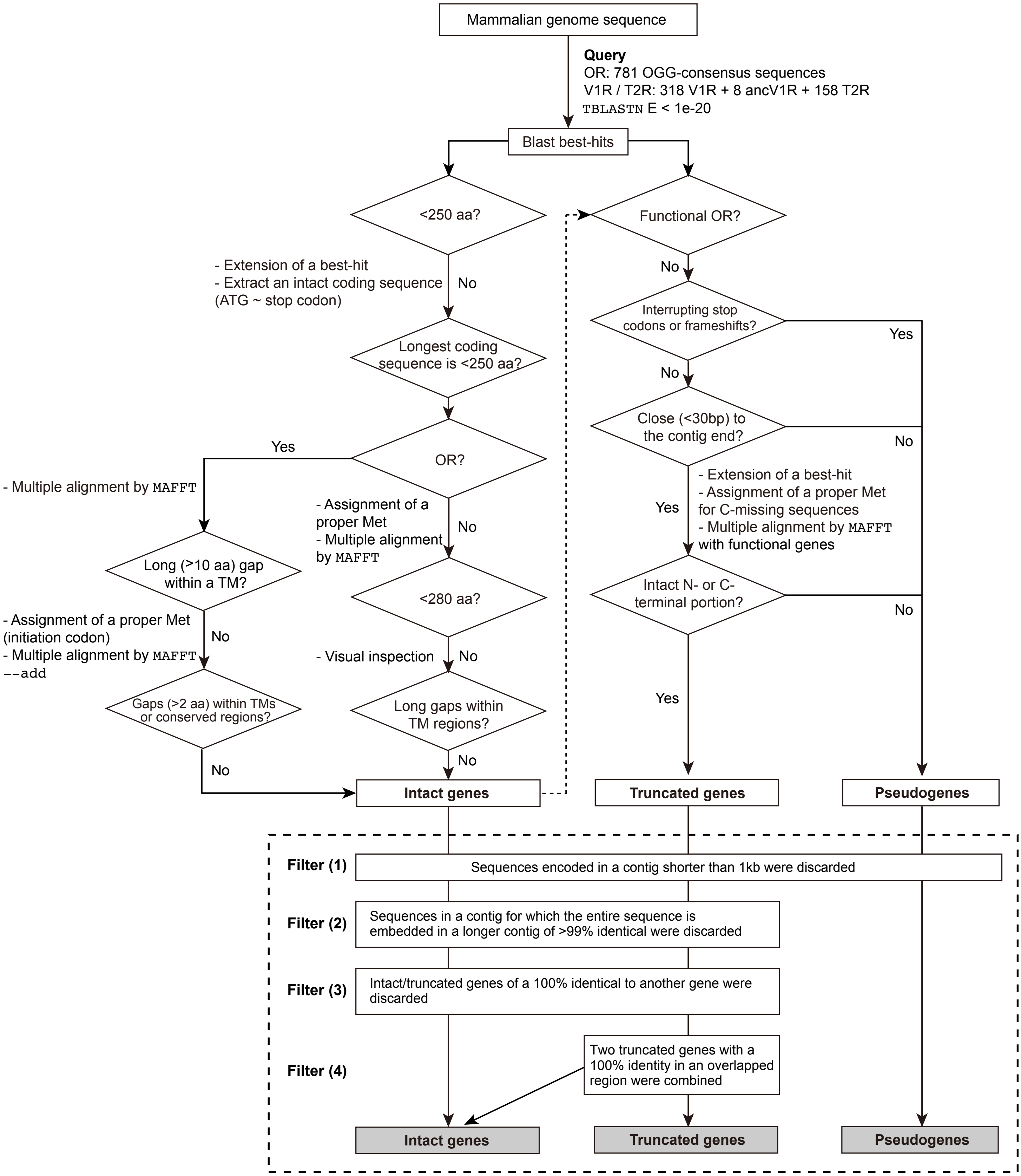

Fig. S18. Flowchart for the identification of OR genes.

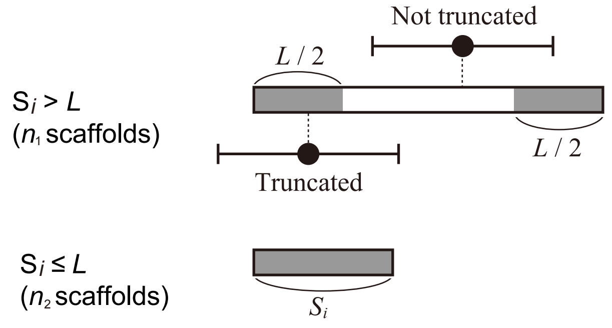

Fig. S19. Estimation of the probability of truncation of V2R genes.

A horizontal line represents a gene with length L, and a black dot representes its center. A rectangular represnts the i-th scaffold with a length of S_i. If the center of a gene is located in a region with gray, the gene is truncated. See Materials and Methods.
