# Supplementary material for: Synchronized Expansion and Contraction of Olfactory, Vomeronasal, and Taste Receptor Gene Families in Hystricomorph Rodents
Source: Mol Biol Evol. 2024 Apr 23;41(4):msae071. doi: 10.1093/molbev/msae071 (PMC11035023; doi:10.1093/molbev/msae071)
Supplement: msae071_Supplementary_Data [file msae071_supplementary_data.zip › Supplementary_Information.pdf]

# Supplementary Information

### Fossil constraints for divergence dating

Fourteen fossil calibrations were used for divergence time analyses corresponding to those selected by Álvarez et. al. (Álvarez et al. 2017) and Verzi et al. (Verzi et al. 2016).

**Bathyergidae** (Álvarez et al. 2017)
*Offset=26.7 Ma, alfa=2.0, beta=1.5 (gamma distribution) (median=29.2 Ma, 97.5% quantile=35.1 Ma).

**Caviidae** (Flynn et al. 1997; Perez and Pol 2012; Upham and Patterson 2015)
*Offset=12.6 Ma, alfa=2.0, beta=3.02 (gamma distribution) (median=17.7 Ma, 97.5% quantile=29.4 Ma).

**Caviomorpha/Phiomorpha** (Antoine et al. 2012; Upham and Patterson 2015)
*Offset=41.0 Ma, alfa=2.0, beta=0.9 (gamma distribution) (median=42.5 Ma, 97.5% quantile=46.0 Ma).

**Caviomorpha** (Antoine et al. 2012; Upham and Patterson 2015)
*Offset=41.0 Ma, M=0, S=0.71 (LogNormal distribution) (median=42.0 Ma, 97.5% quantile=45.0 Ma).

**Cavioidea/Erethizontoidea** (Frailey and Campbell 2004; Bertrand et al. 2012; Vucetich et al. 2015)
*Offset=31.5 Ma, alfa=2.0, beta=2.0 (gamma distribution) (median=34.9 Ma, 97.5% quantile=42.6 Ma).

**Cavioidea** (Bertrand et al. 2012; Álvarez et al. 2017)
*Offset=31.5 Ma, alfa=2.0, beta=2.0 (gamma distribution) (median=34.9 Ma, 97.5% quantile=42.6 Ma).

**Chinchilloidea/Octodontoidea** (Frailey and Campbell 2004; Bertrand et al. 2012; Vucetich et al. 2015)
*Offset=31.5 Ma, alfa=2.0, beta=2.0 (gamma distribution) (median=34.9 Ma, 97.5% quantile=42.6 Ma).

**Chinchillidae** (Bertrand et al. 2012; Álvarez et al. 2017)
*Offset=31.5 Ma, M=0, S=0.91 (LogNormal distribution) (median=32.5 Ma, 97.5% quantile=37.5 Ma).

**Ctenomyidae/Octodontidae** (Kay et al. 1998; Vucetich et al. 2010)
*Offset=25.0 Ma, M=0, S=0.76 (LogNormal distribution) (median=26.0 Ma, 97.5% quantile=29.4 Ma).

**Dolichotinae/Hydrochoerinae** (Álvarez et al. 2017)
*Offset=16.0 Ma, alfa=2.0, beta=2.0 (gamma distribution) (median=19.4 Ma, 97.5% quantile=27.1 Ma)

**Hystricognathi** (Álvarez et al. 2017)
*Offset=42.5 Ma, alfa=2.0, beta=0.9 (gamma distribution) (median=44 Ma, 97.5% quantile=47.5 Ma)

**Hystricomorpha** (Flynn et al. 1986; Marivaux et al. 2004; Sallam et al. 2009; Sallam et al. 2011; Antoine et al. 2012; Upham and Patterson 2015)
*Offset=48.5 Ma, M=0, S=1.46 (LogNormal distribution) (median=49.5 Ma, 97.5% quantile=66.0 Ma).

**Phiomorpha** (Álvarez et al. 2017)
*Offset=32.6 Ma, alfa=2.0, beta=1.3 (gamma distribution) (median=34.8 Ma, 97.5% quantile=39.8 Ma)

**Petromuridae/Thryonomyidae** (Álvarez et al. 2017)
*Offset=17.6 Ma, alfa=2.0, beta=2.0 (gamma distribution) (median=21 Ma, 97.5% quantile=28.7 Ma)

**References:**


Álvarez A, Arévalo RLM, Verzi DH. 2017. Diversification patterns and size evolution in caviomorph rodents. *Biological Journal of the Linnean Society* **121**: 907-922.

Antoine PO, Marivaux L, Croft DA, Billet G, Ganerod M, Jaramillo C, Martin T, Orliac MJ, Tejada J, Altamirano AJ et al. 2012. Middle Eocene rodents from Peruvian Amazonia reveal the pattern and timing of caviomorph origins and biogeography. *Proc Biol Sci* **279**: 1319-1326.

Bertrand OC, Flynn JJ, Croft DA, Wyss AR. 2012. Two New Taxa (Caviomorpha, Rodentia) from the Early Oligocene Tinguiririca Fauna (Chile). *American Museum Novitates* **3750**: 1-36.

Flynn JJ, Guerrero J, Swisher III CC. 1997. Geochronology of the Honda Group. In *Vertebrate Paleontology in the Neotropics- The Miocene fauna of La Venta, Colombia,* (ed. RF Kay, et al.), pp. 44-59. Smithsonian Institution Press.

Flynn JJ, Jacobs LL, Cheema IU. 1986. Baluchimyinae, a new ctenodactyloid rodent subfamily from the Miocene of Baluchistan. *American Museum Novitates* **2841**: 1-58.

Frailey CD, Campbell KE, Jr. 2004. Paleogene rodents from Amazonian Peru: the Santa Rosa Local Fauna. In *The Paleogene Mammalian Fauna of Santa Rosa, Amazonian Peru*, Vol Science Series 40 (ed. KE Campbell, Jr.), pp. 71–130. Natural History Museum of Los Angeles County, Los Angeles.

Kay RF, Macfadden BJ, Madden RH, Sandeman H, Anaya F. 1998. Revised age of the Salla beds, Bolivia, and its bearing on the age of the Deseadan South American Land Mammal "Age". *Journal of Vertebrate Paleontology* **18**: 189-199.

Marivaux L, Vianey-Liaud M, Jaeger J-J. 2004. High-level phylogeny of early Tertiary rodents: dental evidence. *Zoological Journal of the Linnean Society* **142**: 105-134.

Perez ME, Pol D. 2012. Major radiations in the evolution of Caviid rodents: reconciling fossils, ghost lineages, and relaxed molecular clocks. *PLoS One* **7**: e48380.

Sallam HM, Seiffert ER, Simons EL. 2011. Craniodental morphology and systematics of a new family of hystricognathous rodents (Gaudeamuridae) from the late eocene and early oligocene of Egypt. *PLoS One* **6**: e16525.

Sallam HM, Seiffert ER, Steiper ME, Simons EL. 2009. Fossil and molecular evidence constrain scenarios for the early evolutionary and biogeographic history of hystricognathous rodents. *Proc Natl Acad Sci U S A* **106**: 16722-16727.

Upham NS, Patterson BD. 2015. Phylogeny and evolution of caviomorph rodents: a complete phylogeny and timetree for living genera. In *Biology of caviomorph rodents: diversity and evolution,* Vol 1 (ed. VAA D), pp. 63-120. Sociedad Argentina para el Estudio de los Mamíferos (SAREM), Buenos Aires, Argentina.

Verzi DH, Olivares AI, Morgan CC, Álvarez A. 2016. Contrasting Phylogenetic and Diversity Patterns in Octodontoid Rodents and a New Definition of the Family Abrocomidae. *Journal of Mammalian Evolution* **23**: 93-115.

Vucetich MG, Arnal M, Deschamps CM, Pérez ME, Vieytes CE. 2015. A brief history of caviomorph rodents as told by the fossil record. In *Biology of caviomorph rodents:*

*diversity and evolution*, (ed. AI Vassallo, D Antenucci), pp. 11-62. Sociedad Argentina para el Estudio de los Mamíferos (SAREM), Buenos Aires, Argentina.

Vucetich MG, Vieytes EC, Pérez ME, Carlini AA. 2010. The rodents from La Cantera and the early evolution of caviomorphs in South America. In *The Paleontology of Gran Barranca: Evolution and Environmental Change through the Middle Cenozoic of Patagonia*, (ed. RH Madden, et al.), pp. 193-205. Cambridge University Press, New York.

**Absence of the T1R1 gene in the common gundi genome**

Three T1R genes are included within the near-universal single-copy orthologs dataset utilized in BUSCO analysis (see Materials and Methods). Therefore, we first examined the detection status of the T1R genes in the common gundi genome. We found that T1R2 and T1R3 genes were detected, while T1R1 was missing in the common gundi genome (Supplementary Table S5).

We next conducted TBLASTN searches using the T1R1 gene and its flanking genes (*Klhl21*, *Zbtb48*, and *Nol9*) as queries against the whole genome sequences of the species examined with an e-value of 1e-70 (for *Klhl21*, *Zbtb48*, and *T1R1*) or 1e-20 (for *Nol9*). The GRCm38 dataset from UCSC genome browser was employed to ascertain the genes adjacent to the T1R1 gene (Supplementary Fig. S16). As a result, for each of all the species except the common gundi, the T1R1 and its flanking genes were identified from the same scaffold. On the other hand, in the genome of the common gundi, while the flanking genes were identified in one scaffold (PVKB01001179.1), the T1R1 gene was not detected (Supplementary Table S6). We also confirmed that, although there was one assembly gap on the scaffold PVKB01001179.1, the gap was not overlapped to the genomic region of the T1R1 and its flanking genes.

To further examine whether these flanking genes are present in the common gundi genome, we conducted gene prediction for the scaffold PVKB01001179.1. Because no RNA-seq reads are available for the common gundi, we utilized the deep neural network method for calling structural annotation of genes by using Helixer with an option "--lineage vertebrate" (Stiehler et al. 2020). We then performed BLASTP searches using the predicted sequences as queries against the whole GenBank database, and confirmed that each of these sequences showed the highest similarities to *Klhl21*, *Zbtb48*, and *Nol9*. However, we did not identify any sequences showing a similarity to the T1R1 gene (Supplementary Fig. S16).

**References:**

Stiehler F, Steinborn M, Scholz S, Dey D, Weber APM, Denton AK, 2020, Helixer: cross-species gene annotation of large eukaryotic genomes using deep learning, *Bioinformatics*, **36**: 5291-5298.
